# The preventive role of the red gingeng ginsenoside Rg3 in the treatment of lung tumorigenesis induced by benzo(a)pyrene

**DOI:** 10.1038/s41598-023-31710-9

**Published:** 2023-03-20

**Authors:** Jie Xiong, Hongmei Yuan, Shihong Fei, Shengli Yang, Ming You, Li Liu

**Affiliations:** 1 Cancer Center, Union Hospital, Tongji Medical College, Huazhong University of Science and Technology, Wuhan, 430022 China; 2 Institute of Radiation Oncology, Union Hospital, Tongji Medical College, Huazhong University of Science and Technology, Wuhan, 430022 China; 3 Department of Pathology, Wuhan Jinyintan Hospital, Wuhan, 430023 China; 4 Center for Cancer Prevention, Houston Methodist Cancer Center, Houston Methodist Research Institute, Houston, TX 77030 USA

**Keywords:** Cancer, Drug discovery, Plant sciences

## Abstract

Red ginseng has been used in traditional medicine for centuries in Asia. In this study, we evaluated four types of red ginseng grown in different areas (Chinese red ginseng, Korean red ginseng A, Korean red ginseng B, and Korean red ginseng C) for their ability to inhibit lung tumor formation and growth induced by the carcinogen benzo(a)pyrene (B(a)P) in A/J mice and found that Korean red ginseng B was the most effective at lowering the tumor load among the four red ginseng varieties. Moreover, we analyzed the levels of various ginsenosides (Rg1, Re, Rc, Rb2, Rb3, Rb1, Rh1, Rd, Rg3, Rh2, F1, Rk1, and Rg5) in four kinds of red ginseng extract and found that Korean red ginseng B had the highest level of ginsenoside Rg3 (G-Rg3), which suggested that G-Rg3 may play an important role in its therapeutic efficacy. This work revealed that the bioavailability of G-Rg3 was relatively poor. However, when G-Rg3 was coadministered with verapamil, a P-glycoprotein inhibitor, the G-Rg3 efflux in Caco-2 cells was lowered, the small intestinal absorption rate of G-Rg3 in the rat models was increased, the concentration levels of G-Rg3 were elevated in the intestine and plasma, and its tumor-preventive abilities in the tumorigenesis rat model induced by B(a)P were also augmented. We also found that G-Rg3 reduced B(a)P-induced cytotoxicity and DNA adduct formation in human lung cells and rescued phase II enzyme expression and activity through Nrf2 pathways, which may be the potential mechanisms underlying the inhibitory effects of G-Rg3 on lung tumorigenesis. Our study showed a potentially vital role of G-Rg3 in targeting lung tumors in murine models. The oral bioavailability of this ginsenoside was augmented by targeting P-glycoprotein, which allowed the molecule to exert its anticancer effects.

## Introduction

Among the most ubiquitous forms of lung cancer is non-small cell lung cancer (NSCLC), which is one of the leading causes of cancer-related deaths in China and North America^1,2^. A primary factor that increases the risk of NSCLC is smoking cigarettes. The smoke from cigarettes contains more than 60 carcinogens, including benzo(a)pyrene (B(a)P), nitrosamine, and radon decay radioisotopes, to name a few^3^. The polycyclic aromatic hydrocarbon B(a)P is primarily responsible for the toxicity of cigarette smoke. After B(a)P exposure, cellular cytochrome P450 converts it to B(a)P-7,8-dihydrodiol-9,10-epoxide (BPDE), which interacts with DNA to form BPDE-DNA adducts^4^. Moreover, these adducts induce lung tumorigenesis in mice with tumor stages and histopathology analogous to those of human lung tumors^5^. This feature makes the murine B(a)P-induced lung cancer model a pertinent system for evaluating chemical compounds that may have anticancer properties.

A feasible strategy of preventing lung carcinogenesis in high-risk populations, particularly in individuals who smoke cigarettes, is using chemopreventive agents that inhibit the development of intraepithelial neoplastic lesions to circumvent their subsequent advancement to malignancy. A variety of chemopreventive agents have been shown to be effective in animal studies^6^. The promising preventive effect of red ginseng against lung cancer has been highlighted in our earlier report^7^. This herb has been used in Asian traditional medicine for centuries to extend life and health, and the antitumor effects of the herb have also been documented^8^.

The active factors in ginseng are ginsenosides, which are used as compound markers in quality assessments of ginseng extracts. Quantitative analyses of crude ginseng extracts often involve the use of several ginsenosides, including RK1, Rg1, F1, Re, Rb1, Rb2, Rb3, Rd, Rh1, Rh2, Rg3, Rg5, and Rc^9,10^. The clinical application of ginsenosides is rare on account of their very poor oral bioavailability^11^. While the mechanistic aspects of this poor bioavailability are yet to be elucidated, the efflux of ginsenosides by P-glycoprotein (P-gp) may contribute to it^12^. P-gp is one of the most important efflux transporters in the ATP-binding cassette transport superfamily, employing the energy of ATP hydrolysis to release intracellular substances to the external environment. P-gp transporters are usually abundant in the intestine, kidney, liver, and blood‒brain barrier^13^. P-gp is crucially involved in intestinal absorption, and the inhibition of P-gp augments the oral absorption and availability of some anticancer drugs^12,14^. Examples in the literature of previously used inhibitors are verapamil and cyclosporine A^15^. This work involved the establishment of a murine system in which lung cancer was induced by B(a)P to assess the ability of different red ginseng extracts from China and Korea to target malignancies. The extracts were subjected to individual assays for the identification of specific ginsenosides that potentially target carcinogenesis. Then, verapamil was used to target P-gp and increase the oral bioavailability and the therapeutic effect of a cancer-targeting ginsenoside.

The mechanisms by which gisenosides exert their therapeutic effects on carcinogenesis are unknown. Several ginsenosides were found to protect against cellular damage by reducing oxidative stress and activating detoxifying phase II enzymes to reduce carcinogen-induced DNA damage^16^. Glutathione S-transferase (GST) is a typical phase II enzyme that is vitally involved in reducing carcinogen-induced DNA damage^17^. Nuclear erythroid 2-related factor 2 (Nrf2) is an essential transcription factor that regulates redox homeostasis and activates phase II enzyme expression and the cytoprotective antioxidant response^18^. Our study also investigated the effects of the identified ginsenosides on the reduction of cytotoxicity and BPDE-DNA adduct formation induced by B(a)P and the induction of phase II enzymes via the modulation of the Nrf2 pathway in normal lung cells.

## Results

### Reduction of lung tumors by red ginseng extracts in A/J lung cancer model mice

**Figure 1:**
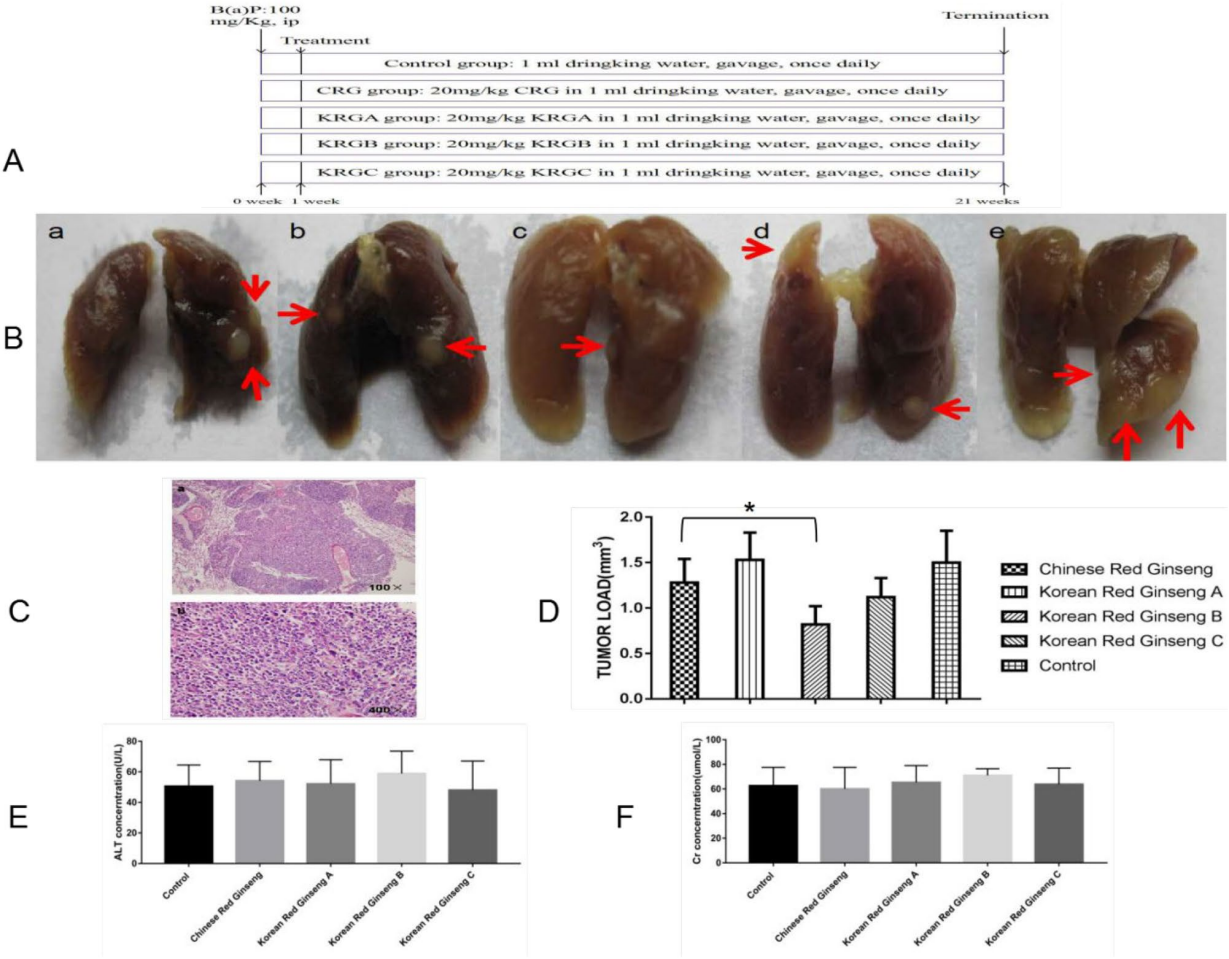
The treatment of lung oncogenesis in A/J mice by red ginseng extracts. (**A**) The experimental design. (**B**) Gross lung tumors in the murine model. Tumors are indicated by arrows. a: Chinese red ginseng group. b: Korean red ginseng A group. c: Korean red ginseng B group. d: Korean red ginseng C group. e: Control group. (**C**) Lung tumors illustrated by light photomicrographs. The magnification is a: 100. b: 400. (**D**) The tumor load in the red ginseng extract groups. (**E**) The plasma levels of the liver enzyme ALT. (**F**) The plasma levels of the kidney enzyme Cr. The data is presented as the mean ± SD. *P < 0.05.

The establishment of the murine B(a)P-induced cancer model was in accordance with previous work^5^. Figure 1A shows the experimental design of the 20-weeks treatment of the B(a)P-induced cancer model mice with water (control), Chinese red ginseng extract (CRG), Korean red ginseng A extract (KRGA), Korean red ginseng B extract (KRGB), and Korean red ginseng C extract (KRGC). The mice were sacrificed by CO_2_ asphyxiation after the 20th week of red ginseng treatment. Figure 1B displays the gross lung tumors in the animals treated with various red ginseng types, and Fig. 1C is a representative light photomicrograph of a tumor sample. The tumor load of KRGB-treated animals was lower (1.5 ± 0.35) than that of the control animals (0.82 ± 0.2, *P* < 0.05), as shown in Fig. 1D. The average tumor load inhibition rate was 45%. These evident alterations in tumor loads were not displayed by the other tested red ginseng extracts (*P* > 0.05). During the 20 weeks of red ginseng treatment, no obvious side effects were noted in the model mice, including no changes in body weight (data not shown) and no liver or kidney toxicity (Fig. 1E,F).

### KRGB possessed the highest ginsenoside Rg3 (G-Rg3) levels compared with those in the other red ginseng extracts

**Figure 2:**
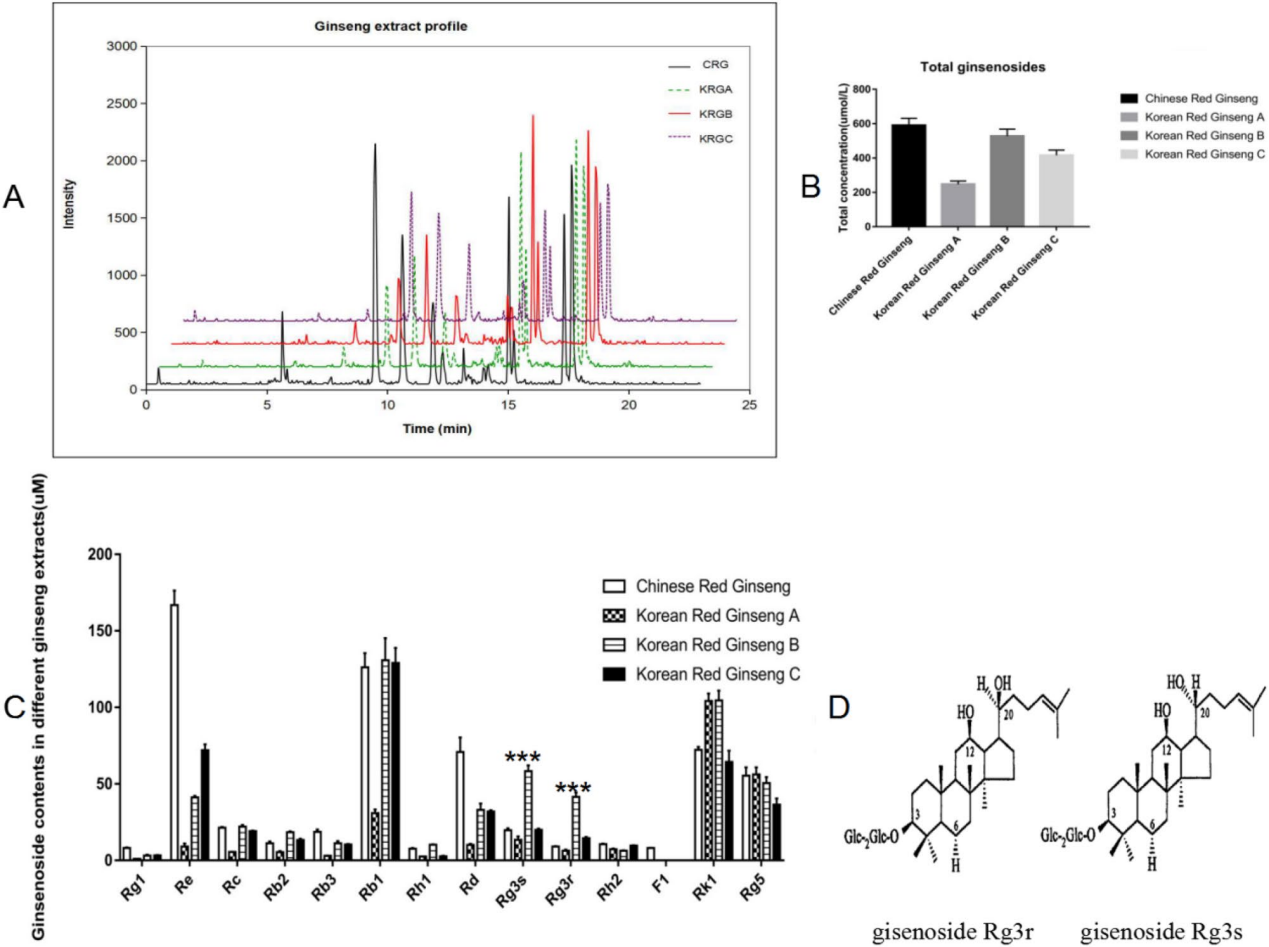
Levels of ginsenosides in the various red ginseng extracts. (**A**) UPLC‒MS/MS chromatogram of four red ginseng extracts. (**B**) Estimation of total ginsenosides in the indicated extracts. (**C**) Detection of individual ginsenosides in the indicated extracts. (**D**) Structures of the ginsenosides G-Rg3r and G-Rg3s stereoisomers. The data is presented as the mean ± SD of triplicate assays. ***P < 0.001.

The red ginseng extracts identified in this work were subjected to ultra-performance liquid chromatography tandem mass spectrometry (UPLC‒MS/MS) analysis to quantify the following ginsenosides: Rg1, Re, Rc, Rb2, Rb3, Rb1, Rh1, Rd, Rg3, Rh2, F1, Rk1, and Rg5. The UPLC and MS conditions for measuring analytes have been described in a previous report^19^. The UPLC‒MS/MS chromatograms of the four red ginseng extracts are depicted in Fig. 2A. There were significant variations in the total level of ginsenosides, and the highest total ginsenoside level (590.27 ± 41.28 µmol/L) was observed in CRG (Fig. 2B). When individual ginsenosides were subject to evaluation (Fig. 2C), KRGB showed the highest level of G-Rg3 (58.33 ± 3.81 µmol/L for G-Rg3s and 41.56 ± 2.88 µmol/L for G-Rg3r) compared to that in other red ginseng types (*P* < 0.001). G-Rg3 occurs as a pair of stereoisomers, G-Rg3r and G-Rg3s, which differ in the position of a hydroxyl group on carbon-20 (Fig. 2D). The results suggest that G-Rg3r or G-Rg3s may have vital anti-oncogenic potential in B(a)P-induced cancer model mice.

### Low in vivo ginsenoside oral bioavailability

The UPLC‒MS/MS study entailed the quantification of ginsenosides in the intestine and blood samples following the 20-weeks treatment. KRGB treatment revealed the presence of 0.0063 ± 0.0005 µg/mL of only Rg5 in the blood. The lack of detection of the remaining ginsenosides is indicative of the poor oral bioavailability and hence decreased exposure levels of these ginsenosides.

### Verapamil administration augmented G-Rg3 Caco-2 transcellular transport

**Figure 3:**
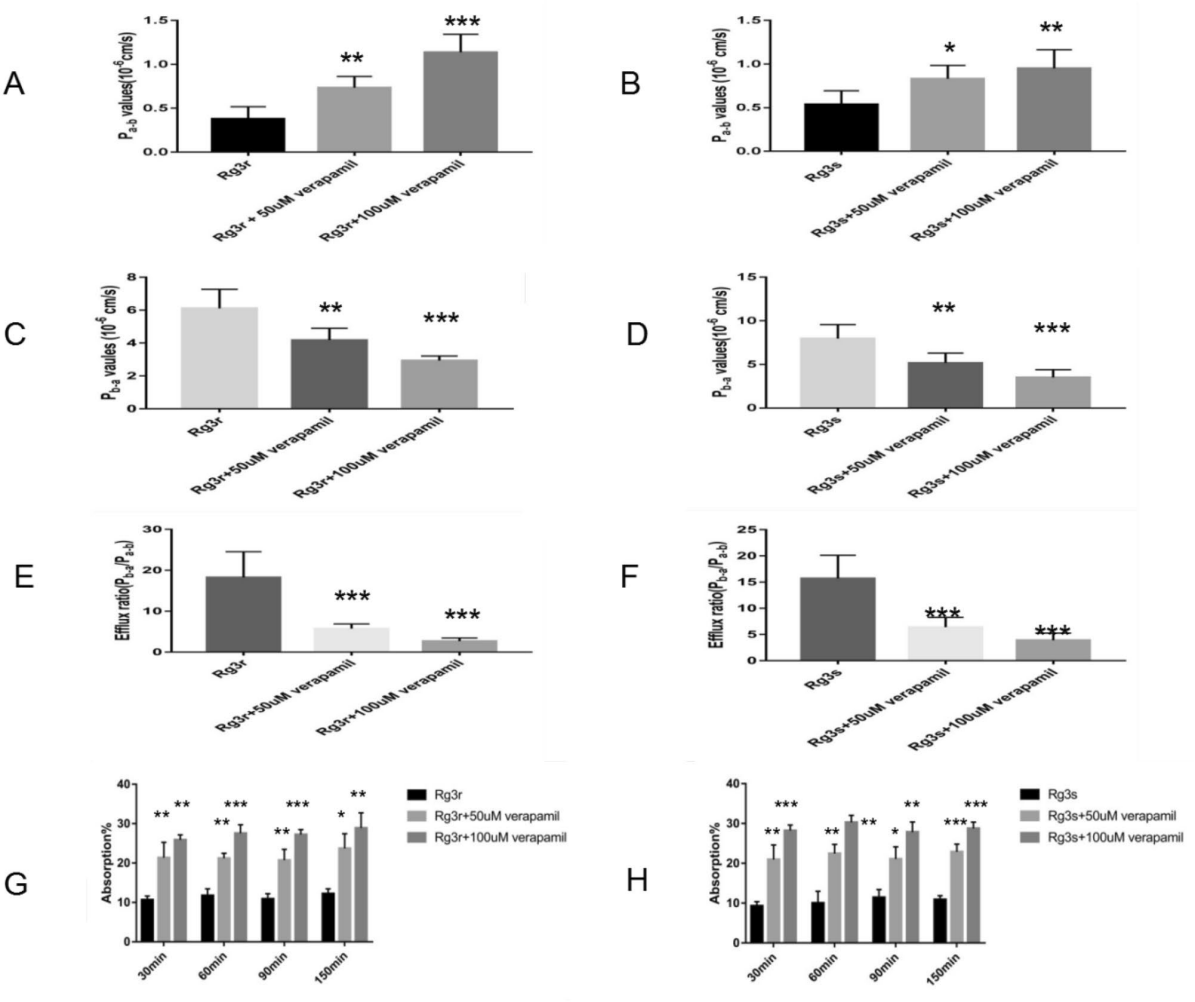
G-Rg3 transcellular transport in the Caco-2 monolayer and intestinal absorption in the rat perfusion assay. (**A**) Pa-b values of G-Rg3r groups in the Caco-2 monolayer. (**B**) Pa-b values of G-Rg3s groups in the Caco-2 monolayer. (**C**) Pb-a values of G-Rg3r groups in the Caco-2 monolayer. (**D**) Pb-a values of G-Rg3s groups in the Caco-2 monolayer. (**E**) Efflux ratio of G-Rg3r groups in the Caco-2 monolayer. (**F**) Efflux ratio of G-Rg3 groups in the Caco-2 monolayer. (**G**) Intestinal absorption percentages of G-Rg3r in the rat perfusion assay. (**H**) Intestinal absorption percentages of G-Rg3s in the rat perfusion assay. Comparisons of permeability and absorption were performed with no verapamil added. The data is presented as the mean ± SD of five independent experiments. *P < 0.05, **P < 0.01, ***P < 0.001.

The morphology and biochemistry of the Caco-2 colon adenocarcinoma cell line are similar to those of human enterocytes, demonstrating its utility in evaluating transport across intestinal cells with regard to the intestinal epithelial barrier. This assay was performed in accordance with an earlier study^20^. Figure 3A,B,C,D,E,F are representative images of G-Rg3r and G-Rg3s transcellular transport using the Caco-2 monolayer model. Transcellular transport of G-Rg3r or G-Rg3s across the Caco-2 monolayer from the basolateral to the apical side (*P*b-a) was significantly higher than that from the apical to the basolateral side (*P*a-b). For G-Rg3r, the average *P*a-b was 0.38 ± 0.06, which increased to 0.73 ± 0.06 after 50 µmol/L verapamil treatment and 1.14 ± 0.09 after 100 µmol/L verapamil treatment (*p* < 0.01 and 0.001, respectively; Fig. 3A). The observations for G-Rg3s followed a similar pattern (Fig. 3B) and the results indicated that verapamil treatment augmented G-Rg3r and G-Rg3s transport. Verapamil treatment also resulted in a significant decrease in the average *P*b-a and efflux ratios of G-Rg3r and G-Rg3s (Fig. 3C,D,E,F), indicating that verapamil treatment decreased the efflux of ginsenosides in Caco-2 cells.

### Verapamil treatment augmented G-Rg3 intestinal absorption

In accordance with earlier work^20^, in situ rat intestinal perfusion was performed to ascertain if the absorption of G-Rg3 in the intestine was increased after verapamil treatment. Figure 3G,H show representative perfusion assays assessing the intestinal absorption percentages of G-Rg3r and G-Rg3s over the aforementioned time periods in cancer model rats. The initial weak G-Rg3r absorption percentage of approximately 10% was elevated to more than 20% after 50 µM verapamil treatment and more than 25% after 100 µM verapamil treatment. Similarly, the initial absorption rate of 10% for G-Rg3s also showed a spike to more than 20% after 50 µM verapamil treatment and close to 30% after 100 µM verapamil treatment, which indicated that treatment with the P-gp inhibitor verapamil augmented the intestinal G-Rg3 absorption in lung cancer model mice.

### Verapamil treatment improved the cancer preventive effect of G-Rg3 in cancer model mice

**Figure 4:**
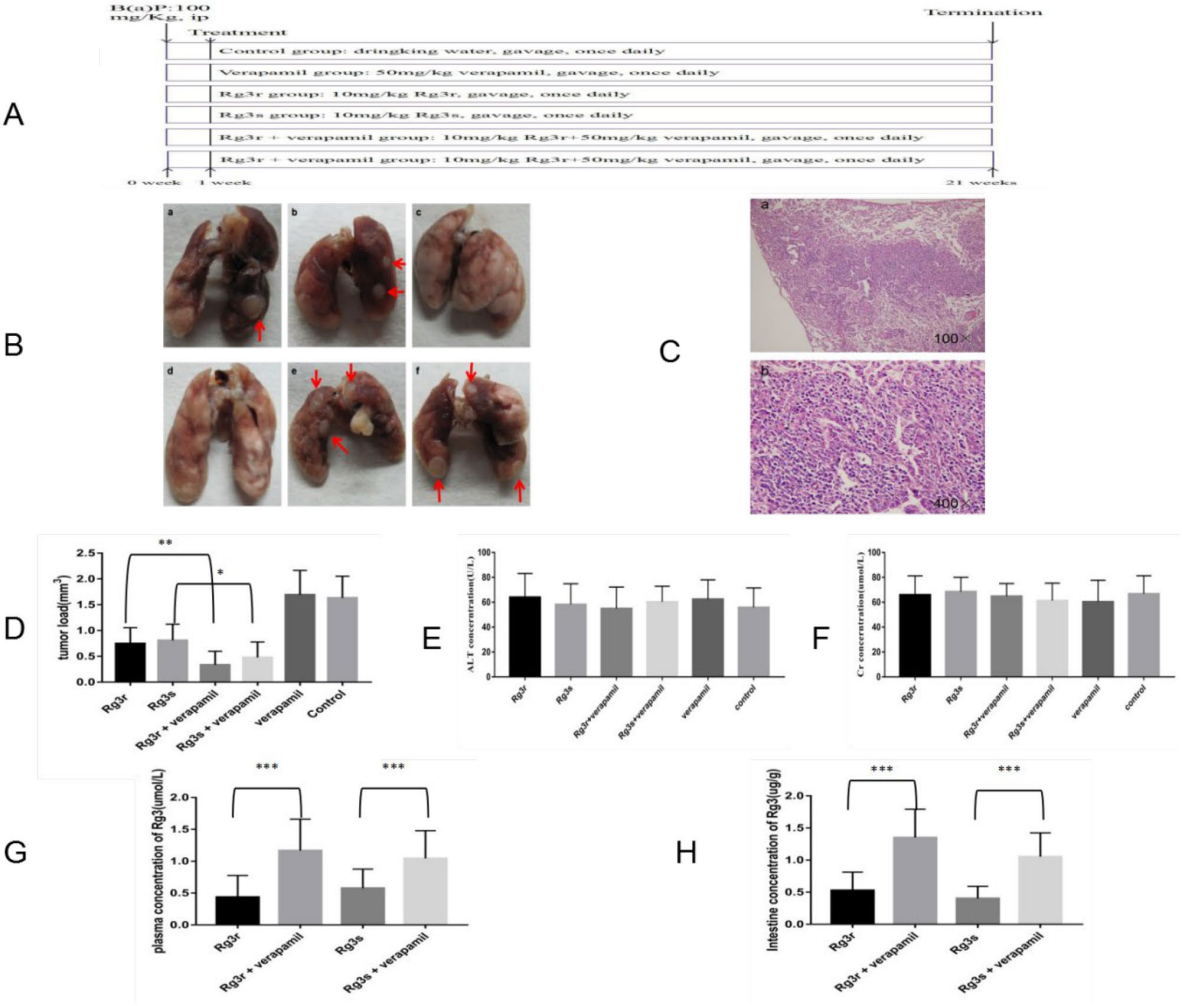
The tumor load after G-Rg3 treatment and the levels of G-Rg3r and G-Rg3s in the plasma or intestine in the indicated groups. (**A**) Experimental design. (**B**) Gross tumors in the murine model. Tumors are indicated by arrows. a: G-Rg3r. b: G-Rg3s. c: G-Rg3r combined with verapamil. d: G-Rg3s combined with verapamil. e: Verapamil. f: Control. (**C**) Light photomicrographs of tumors with magnification at. a: 100X. b: 400X. (**D**) G-Rg3 + verapamil treatment on tumor load in A/J mice. (E) The plasma levels of the liver enzyme ALT. (F) The plasma levels of the kidney enzyme Cr. (G) Levels of G-Rg3r or G-Rg3s in the plasma of the indicated groups. (H) Levels of G-Rg3r or G-Rg3s in the intestine of the indicated groups. The data is presented as the mean ± SD of triplicate assays. *P < 0.05, **P < 0.01, ***P < 0.001.

In accordance with the approach discussed above, the random assignment of the B(a)P-induced cancer model mice into six groups is represented in Fig. 4A. No significant body weight loss or clinical signs of toxicity were observed in the G-Rg3 treatment groups compared with the control group (data not shown). Following the 20-weeks treatment, the lungs of each mouse were collected. Figure 4B shows the gross lung tumors of mice in the aforementioned treatment groups, and Fig. 4C is representative light photomicrograph of the indicative tumors. With regard to the tumor loads across the groups (Fig. 4D), the values were 0.75 ± 0.29 mm^3^ and 0.81 ± 0.30 mm^3^ for the G-Rg3r-treated and G-Rg3s-treated mice, respectively, against 1.63 ± 0.40 mm^3^ for the control mice (*p* < 0.001), suggesting that G-Rg3 treatment decreased the tumor load of mice. This decrease was further augmented by verapamil adminstration, with the values falling from 0.75 ± 0.29 mm^3^ to 0.33 ± 0.25 mm^3^ for verapamil + G-Rg3r-treated mice (*p* < 0.01) and from 0.81 ± 0.30 mm^3^ to 0.29 ± 0.21 mm^3^ verapamil + G-Rg3s-treated mice (*p* < 0.05), which is indicative of the ability of verapamil to increase the inhibitory effect of G-Rg3 on tumorigenesis. The tumor load among the control versus verapamil, G-Rg3r versus G-Rg3s, and verapamil + G-Rg3r versus verapamil + G-Rg3s sets displayed no obvious differences. Additionally, there were no obvious liver and kidney toxicity effects associated with the treatments that were evaluated (Fig. 4E,F).

### Verapamil treatment increased G-Rg3 levels in the intestines and plasma of model mice

In accordance with the approach described in the methods section, G-Rg3 levels in the B(a)P-induced cancer model mice were assessed by UPLC‒MS/MS once the 20-weeks treatment period was completed. Figures 4G,H are show the levels of G-Rg3 in the plasma and intestine, respectively. The G-Rg3r plasma level was 0.44 ± 0.32 µmol/L, which was increased to 1.17 ± 0.47 µmol/L (*p* < 0.001) when verapamil was coadministered, while in the intestine, the G-Rg3r level of 0.53 ± 0.08 µg/g was increased to 1.35 ± 0.13 µg/g (*p* < 0.001) when verapamil was coadministered. For G-Rg3s, the results followed a similar pattern, indicating that verapamil treatment augmented the oral bioavailability of G-Rg3 in A/J model mice.

### G-Rg3 treatment attenuated B(a)P-induced cytotoxicity in human embryonic lung (hEL) cells

**Figure 5:**
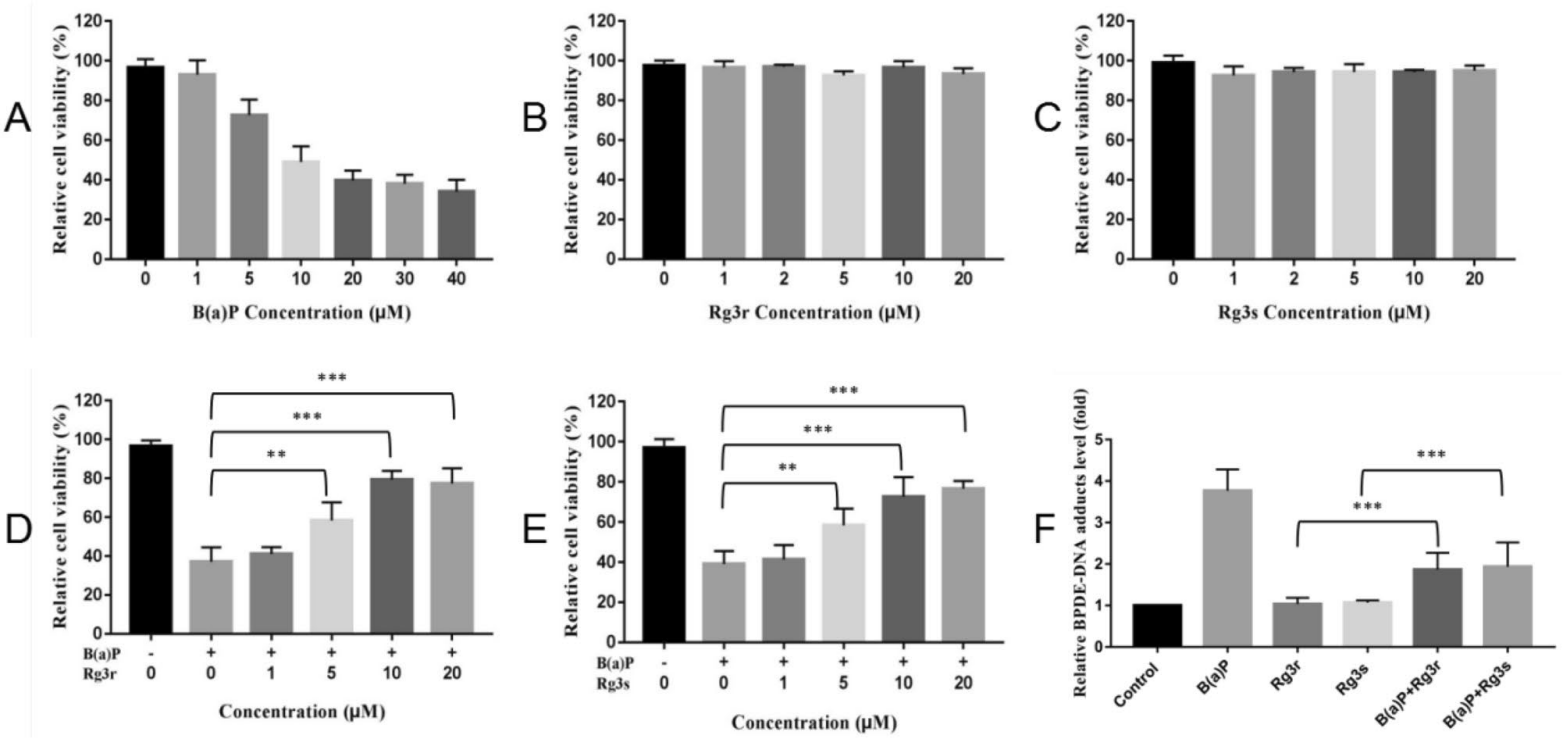
Cell viability and BPDE-DNA adduct formation in hEL cells treated with G-Rg3 and B(a)P. (**A**) Cell viability of hEL cells treated with B(a)P. (**B**) Cell viability of hEL cells treated with G-Rg3r. (**C**) Cell viability of hEL cells treated with G-Rg3s. (**D**) Cell viability of hEL cells treated with B(a)P and G-Rg3r. (**E**) Cell viability of hEL cells treated with B(a)P and G-Rg3s. (**F**) BPDE-DNA adduct levels of hEL cells treated with B(a)P and G-Rg3. The data is presented as the mean ± SD of triplicate assays. *P < 0.05, **P < 0.01, ***P < 0.001.

Cell viability assays were used to evaluate the cytotoxicity of B(a)P and G-Rg3 on hEL cells. B(a)P-induced cytotoxicity in hEL cells is shown in Fig. 5A, while the nontoxic nature of G-Rg3r and G-Rg3s is shown in Figs. 5B,C. To evaluate the cytoprotective effects of G-Rg3, B(a)P was coadministered to hEL cells along with various concentrations of G-Rg3r or G-Rg3s. As shown in Fig. 5D, G-Rg3r at concentrations of 5 µM, 10 µM, and 20 µM restored cell viabilities to 58.3%, 79.3%, and 77.3%, respectively. Similar results could be seen in the G-Rg3s group, with the cell viabilities restored to 58.3%, 72.7%, and 76.7% by G-Rg3s concentrations of 5 µM, 10 µM, and 20 µM, respectively (Fig. 5E). The presence of BPDE-DNA adducts was measured by using an ELISA kit. Our results showed that the B(a)P treatment group had increased BPDE-DNA adduct levels compared to those in the control group, but that B(a)P cotreatment with G-Rg3 significantly decreased the BPDE-DNA adduct level compared to that of B(a)P treatment alone, as shown in Fig. 5F (1.87 ± 0.33 vs. 3.77 ± 0.42 for G-Rg3r, and 1.93 ± 0.48 vs. 3.77 ± 0.42 for G-Rg3s, *p* < 0.001).

### Modulatory effect of G-Rg3 on the expression of GST

**Figure 6:**
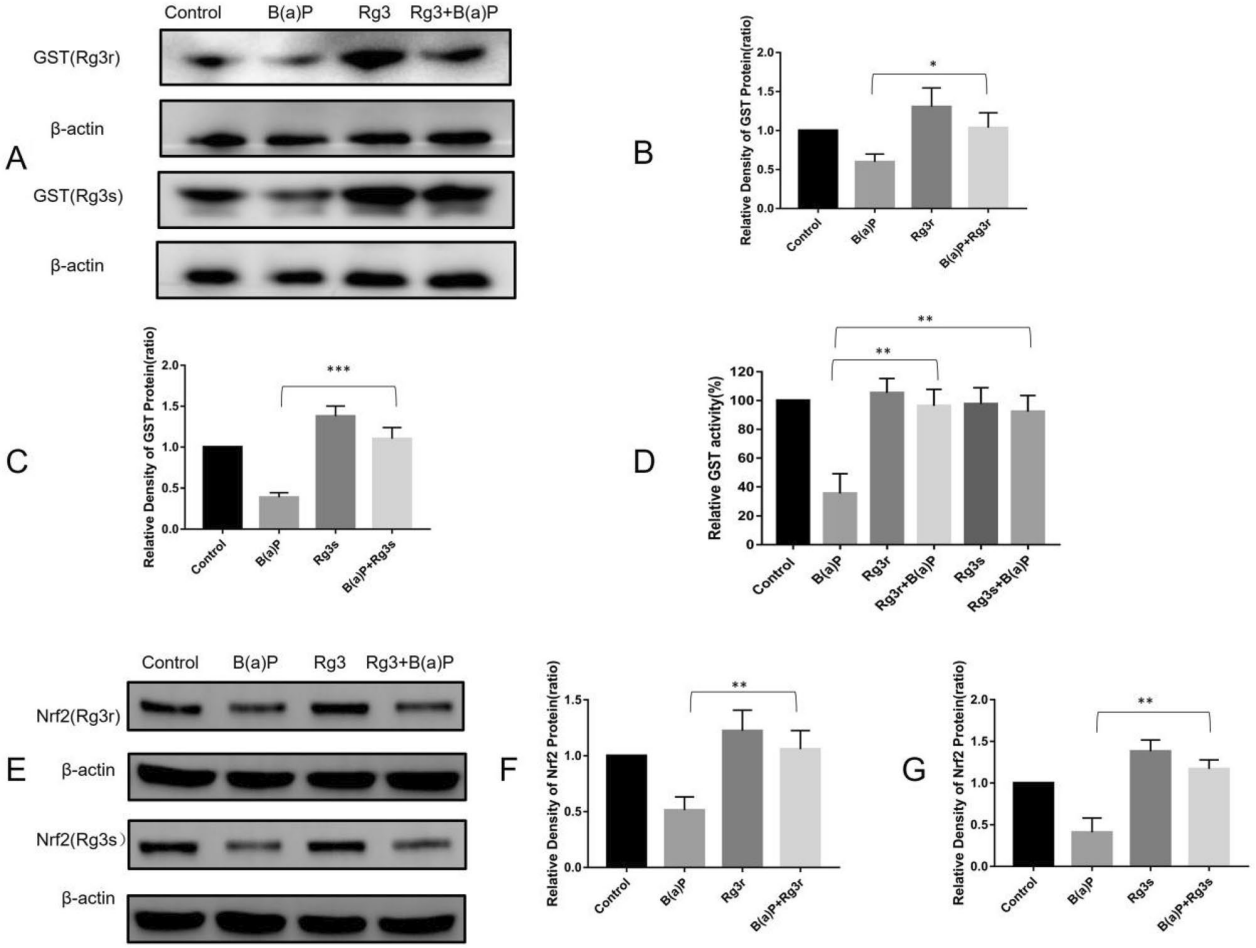
Expression of GST and Nrf2 in hEL cells treated with B(a)P and G-Rg3. (**A**) GST expression was detected by western blotting. (**B**) Quantitative GST expression in hEL cells treated with B(a)P and G-Rg3r. (**C**) Quantitative GST expression in hEL cells treated with B(a)P and G-Rg3s. (**D**) GST activity of hEL cells treated with B(a)P and G-Rg3. (**E**) Nrf2 expression detection by western blotting. (**F**) Quantitative Nrf2 expression in hEL cells treated with B(a)P and G-Rg3r. (**G**) Quantitative Nrf2 expression in hEL cells treated with B(a)P and G-Rg3s. The data is presented as the mean ± SD of triplicate assays. *P < 0.05, **P < 0.01, ***P < 0.001.

GST enzyme expression was detected after cotreatment with 10 µM B(a)P and with 10 µM G-Rg3r or G-Rg3s. Our results showed that B(a)P downregulated GST expression (59.7 ± 8.2% in G-Rg3r and 39 ± 4.5% in G-Rg3s groups), and B(a)P cotreatment with G-Rg3r or G-Rg3s recovered the expression of GST (103.7 ± 15.5% in G-Rg3r and 110 ± 11.1% in G-Rg3s groups, *p* < 0.05 and *p* < 0.001, respectively, Figs. 6A,B,C). GST activity was evaluated using an activity assay kit. Our results showed that the cotreatment group had higher GST activity compared with that in the B(a)P only group (96.3 ± 6.6% vs. 35.7 ± 7.8% in the G-Rg3r group, and 92.3 ± 6.5% vs. 35.7 ± 7.8% in the G-Rg3s group, *p* < 0.001, Fig. 6D).

### Modulatory effect of G-Rg3 on Nrf2 pathway expression

To elucidate the pathways involved in the G-Rg3-mediated suppression of B(a)P-induced carcinogenesis, Nrf2 expression was assessed using western blotting. As shown in Fig. 6E,F,G, the B(a)P only treatment group showed a decreased level of Nrf2 compared with that of the control group; however, the B(a)P-G-Rg3 group showed an increased level of Nrf2 compared with that of the B(a)P treatment group (106 ± 9.5% vs. 51.3 ± 6.8% for G-Rg3r, and 117 ± 6.2% vs. 41 ± 9.8% for G-Rg3s, *p* < 0.01).

### Reduction of BPDE-DNA Adducts by G-Rg3 is Dependent on Nrf2

**Figure 7:**
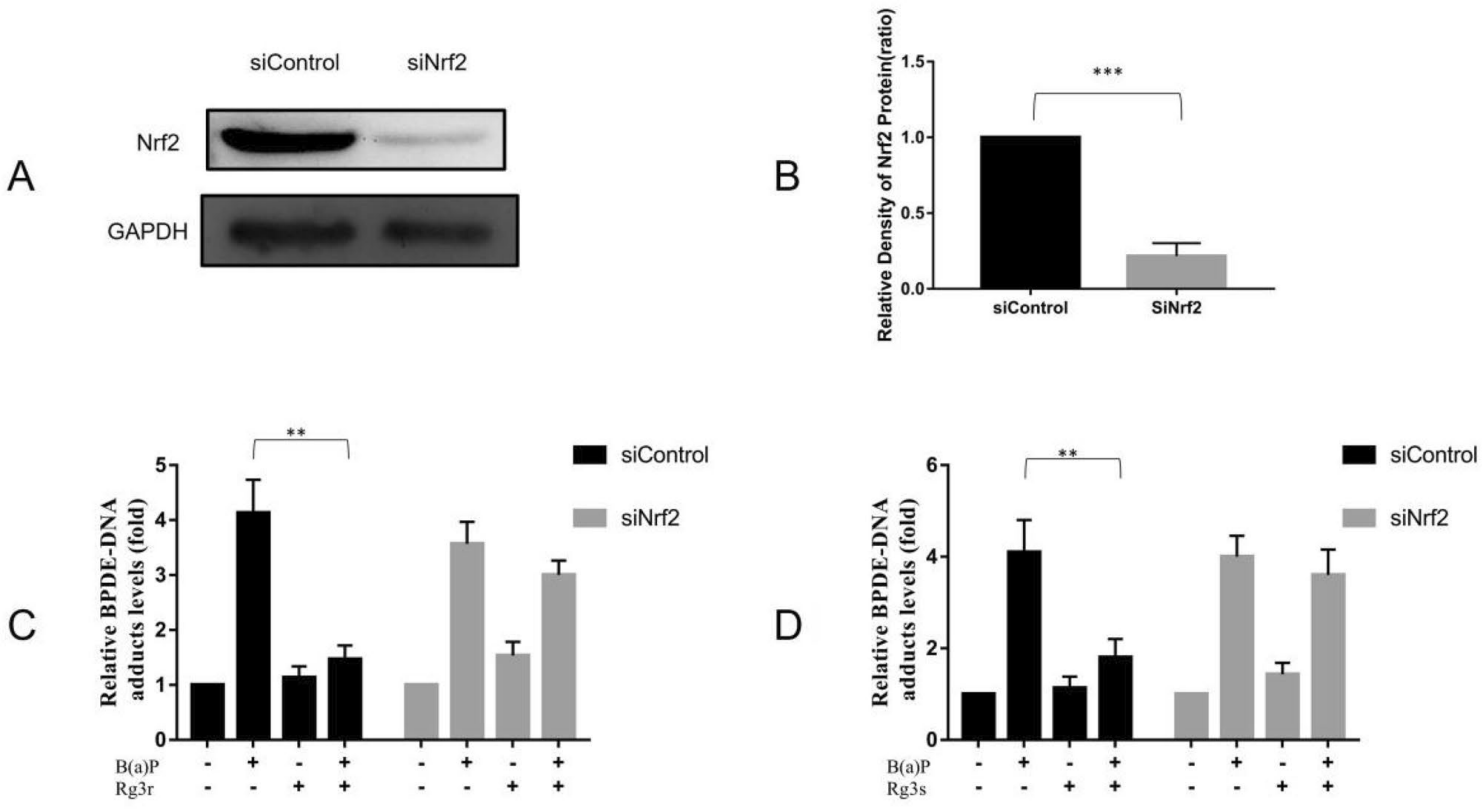
Effect of Nrf2 knockdown on BPDE-DNA adduct formation in hEL cells. (**A**) The knockdown of Nrf2 was confirmed by western blotting. (**B**) Quantification of the Nrf2 band intensity. (**C**) Effect of Nrf2 knockdown on BPDE-DNA adduct levels in hEL cells treated with B(a)P and G-Rg3r. (**D**) Effect of Nrf2 knockdown on BPDE-DNA adduct levels in hEL cells treated with B(a)P and G-Rg3s. The data is presented as the mean ± SD of triplicate assays. *P < 0.05, **P < 0.01, ***P < 0.001.

We confirmed the preventive effect of Nrf2 using specific small interfering RNA (siRNA), which was used to knockdown Nrf2 expression. Knockdown of Nrf2 was confirmed by western blotting (Fig. 7A,B). As shown in Fig. 7C,D, compared with B(a)P treatment alone in the control siRNA group, cotreating hEL cells with B(a)P and G-Rg3 resulted in a reduction in BPDE-DNA adducts (1.47 ± 0.21 vs. 4.13 ± 0.49 for G-Rg3r, and 1.8 ± 0.32 vs. 4.1 ± 0.57 for G-Rg3s, *p* < 0.01). However, this inhibitory effect of G-Rg3 on BPDE-DNA formation was eliminated by Nrf2 knockdown. There were no significant differences in BPDE-DNA adduct formation between B(a)P and G-Rg3 cotreatment and B(a)P treatment alone in the siNrf2 group (3.0 ± 0.21 vs. 3.56 ± 0.32 for G-Rg3r, and 3.6 ± 0.45 vs. 4.0 ± 0.37 for G-Rg3s, *p* > 0.05).

## Discussion

This study assessed the preventive effect of various red ginseng extracts on B(a)P-induced lung cancer model mice, and treatment with KRGB significantly reduced the tumor load. Given that G-Rg3 was found to be present at its highest level in this ginseng extract among the others, the vital involvement of this ginsenoside in suppressing tumorigenesis was explored. Both G-Rg3r and G-Rg3s, which are the two epimers of G-Rg3, significantly decreased the tumor load in the B(a)P-induced cancer mode mice. G-Rg3r and G-Rg3s exert anticancer effects by inducing tumor cell apoptosis^21^ suppressing tumor growth^22^, arresting the cell cycle^23^, and targeting angiogenesis^24^. G-Rg3 has also been shown to inhibit the ability of cells to metastasize^25^ and the ability of G-Rg3 to enhance the effects of chemotherapy and radiotherapy^26,27^ have also been documented. The reduction of the genotoxic effects of B(a)P by G-Rg3s treatment has been shown by Poon et al.^28^. This research suggests the therapeutic potential of G-Rg3 in combatting environmental carcinogenic molecules and preventing cancer.

Although ginsenosides have promising preventive potential, the poor oral bioavailability of ginsenosides is a challenge for utilizing these molecules clinically. Pharmacokinetic analyses of orally administered ginsenosides in rats revealed that the bioavailability remained below 5%^29^. These analyses revealed a low level of only Rg5 in the blood after a 20-weeks treatment period. While the underlying mechanisms of this poor bioavailability remain to be elucidated, the involvement of P-gp in the efflux of ginsenosides is hypothesized. This work demonstrated for the first time that adminstration of verapamil, a P-gp blocker, augmented G-Rg3r and G-Rg3s oral bioavailability. Thus, this finding suggests that G-Rg3r and G-Rg3s function as P-gp substrates, which regulates their efflux.

This work demonstrated that verapamil cotreatment augmented G-Rg3 oral bioavailability in a lunger cancer mouse model. This finding was supported by the augmentation of G-Rg3 intestinal transcellular transport to increase its absorption as a result of blocking P-gp. The Caco2 cell-based assay demonstrated that verapamil treatment reduced G-Rg3r and G-Rg3s efflux while improving the membrane permeability. Research by Yang et al. revealed that the treatment of cyclosporine A (another P-gp blocker) increased the bioavailability of ginsenoside Rh2 to more than 30% from an initial value of 1%^20^. Compound K and Rg1 ginsenosides have also shown similar results^30,31^. Compound K efflux in Caco-2 cells was evidently reduced to less than 3 from 26.6, while its intracellular levels spiked by a factor of 40 when both verapamil and cyclosporine A were used^30^. Rg1 levels were increased in rat pulmonary epithelial cells in the presence of verapamil, indicative of the function of P-gp in ginsenoside efflux, as shown by Meng et al.^31^. However, verapamil did not exhibit the same effect on the efflux of a few ginsenosides, such as Rg1, F1, Rh1, and Re, showing that they are not targeted by P-gp substrates, as shown by Liang et al.^32^. This observation is potentially due to the involvement of other transporters and alternative ginsenoside structures.

The mechanisms underlying to the cancer-preventive effect of G-Rg3 are unknown. Previous studies showed that G-Rg3 protected against DNA damage and cell apoptosis by reducing both oxidative stress and inflammation^16,33^, which may be potential mechanisms of its preventive effect on B(a)P-induced tumorigenesis. Several reports showed that B(a)P-induced genotoxicity was reduced by phase II enzyme modulation before BPDE-DNA formation^34^. GST is a typical phase II enzyme and reduces B(a)P-induced DNA damage by promoting GSH conjugation with BPDE to inhibit BPDE-DNA adduct formation^35^. Our results showed that G-Rg3 treatment decreased cytotoxicity and BPDE-DNA adduct formation induced by B(a)P in hEL cells and rescued GST expression and activity in vitro. However, these effects were nonexistent in the absence of Nrf2, which suggested that G-Rg3 elicits a cytoprotective effect through the Nrf2 pathway. Nrf2 is the major transcription factor of phase II detoxification enzymes that promote the elimination of xenobiotics^36^. Activation of the Nrf2 pathway induces cytoprotection and reduces tissue injury^37^. Moreover, several reports have substantiated the tumor suppressor effect of Nrf2 in carcinogenesis^38^. Our studies indicated that the induction of the Nrf2 pathway by G-Rg3 played an important regulatory role against B(a)P-induced genotoxicity, which caused B(a)P detoxification through the activation of phase II enzymes to inhibit the tumorigenesis process.

## Conclusions

Our work unveiled the potential of red ginseng in preventing lung cancer induced by B(a)P in a murine system through the vital involvement of the ginsenoside G-Rg3. The poor oral bioavailability of this molecule hinders its clinical utility. However, this study is the first to reveal that G-Rg3 acts as a substrate for P-gp and that the adminstration of P-gp inhibitors augments the bioavailability of G-Rg3 in vitro and in vivo. G-Rg3 reduced B(a)P-induced cytotoxicity by modulating Nrf2 pathways, which may be a potential mechanism of its preventive function. Our study supports the possible use of ginsenoside G-Rg3 for the prevention and therapy of lung cancer.

## Materials and methods

### Mice, cell lines and reagents

Six-week-old female A/J mice (20 ± 1 g) and 7-weeks-old male Wistar rats (250 ± 20 g) were obtained from the Jackson Laboratory (Bar Harbor, USA) and the animal institution of Wuhan University (Wuhan, China), respectively. The China Center for Type Culture Collection (Wuhan, China) provided us with Caco-2 and hEL cells. Sigma‒Aldrich (St. Louis, USA) was the source of B(a)P and tricaprylin. Purified ginsenosides G-Rg3r and G-Rg3s, dimethyl sulfoxide (DMSO), the CellTiter-96 proliferation assay (MTS) kit, verapamil, minimum essential medium (MEM), and fetal bovine serum (FBS) were commercially ordered from Chengdu Must Bio-Technology Co., Ltd. (Chengdu, China). The QIAamp DNA Mini Kit and BPDE-DNA adduct ELISA kit were purchased from Qiagen (Stanford, CA, USA) and Cell Biolabs (San Diego, CA, USA). The GST activity assay kit and the total protein assay kit (with standard BCA method) were purchased from Solarbio (Beijing, China). All the red ginseng extracts were stored in Ming You’s lab^7^. The Hong Kong Baptist University (Hongkong, China) and the Korea Cancer Center Hospital (Seoul, South Korea) were the commercial sources of CRG extract and various red ginseng extracts of different Korean origin, including KRGA, KRGB, and KRGC. Red ginseng were manufactured from roots of 6-years-old fresh ginseng. Red ginseng extract was prepared by washing ginseng with water three times, and then the water extracts were concentrated and finally dried at low temperature to produce ginseng extract powder. Antibodies (anti-Nrf2, anti-GST, and β-actin), horseradish peroxidase-conjugated anti-rabbit immunoglobulin G (IgG), transfection reagent, control siRNA, and Nrf2-siRNA were purchased from Santa Cruz Biotechnology (Santa Cruz, CA, USA).

### Cell culture

Caco2 and hEL cells were cultured in a 100 mm^2^ cell culture dish with MEM containing 10% FBS at 37 °C in a humidified atmosphere of 5% CO_2_. To determine the effects of the treatment conditions, hEL cells were incubated with various concentrations of B(a)P and G-Rg3 in MEM for 48 h. The cells were subjected to further analysis or harvested to prepare cell-free extracts.

### Establishment of the mouse lung cancer model

All experiments were approved by the laboratory animal ethics committee of Tongji Medical College, Huazhong University of Science and Technology (approval no. 2019; file no. 4587TH). All experiments were performed in accordance with relevant guidelines and regulations, and the study was conducted in accordance with Animal Research: Reporting of in vivo Experiments (ARRIVE) guidelines. Eight-week-old A/J mice were given an initial intraperitoneal injection of B(a)P in tricaprylin (100 mg/kg in 0.2 mL). One week later, the mice were randomly divided into control and different treatment groups with 15 mice per group, and treatment was performed once every day via oral gavage. Following 20 weeks of treatment, the animals were sacrificed by CO_2_ asphyxiation. The lungs were collected and fixed for 24 h. Quantification of the surface tumor count and individual tumor size on each lung was performed under a dissecting microscope. The tumor volume (V) estimation was calculated using the following expression: V (mm^3^) = 4/3πr^3^, where r is the tumor diameter. The net sum of all the volumes of tumors in a mouse lung was the total tumor volume, with the mean total tumor volume per group represented the tumor load. Whole blood and intestine samples were collected and stored at − 80 °C for UPLC‒MS/MS assays. Serum was collected and analyzed for alanine transaminase (ALT) and serum creatinine (Cr) levels by an automatic biochemical analyzer to evaluate liver and kidney function.

### Processing samples and UPLC‒MS/MS analysis

As described above, the collected samples were retrieved from cold storage, thawed, weighed, and placed in a test tube. To this, 0.5 µM phloridzin (internal standard) in 0.8 mL of methanol solution was added. Then, the tissue was homogenized using a Tissue-Tearor and the homogenate was subsequently transferred into a 1.5 mL microcentrifuge tube. The mixture was centrifuged for 15 min at 15,500 rpm. Following the removal of 1.0 mL of the supernatant, nitrogen–based drying was performed. Two hundred microliters of methanol was used for reconstitution. Blood was collected and processed on the same lines and used as a reference for all measurements.

### Transcellular transport study

A Transwell 24-well plate was seeded with 1.0 × l0^5^ Caco-2 cells per well to evaluate the potential augmentation of G-Rg3 transport due to the addition of verapamil. The cells were subjected to washing using HBSS and preincubation at 37 °C following 3 weeks of culture. The basolateral or apical side of the monolayer received 400 µL of 10 µM G-Rg3 (G-Rg3r, G-Rg3s, or the mix along with 50 or 100 µM verapamil), while 600 µL of HBSS solution was added to the other side. Collection of 100 µL medium was performed at the indicated times (0, 15, 30, 45, 60, 90, and 120 min), and 100 µL HBSS was added to replenish this volume. These samples were stored at − 4 °C until UPLC‒MS/MS assays. The expression Papp = dQ/(dT × A × C0) was utilized to quantify the apparent unidirectional permeability for both the apical and basolateral sides and vice versa (Pa-b and Pb-a, respectively); dQ/dT represents the concentration change, A (0.6 cm^2^) represents the monolayer surface area, and C0 represents the initial donor concentration. The efflux ratio was calculated as Pb-a/Pa-b, which represents the degree of efflux transport of the study drug.

### In situ mouse intestinal perfusion study

Male Wistar rats that were subjected to fasting with access to only water for 24 h were anesthetized by intravenous injection of 3.5% pentobarbital solution. The silicone tube for cannulation at the duodenum end was the inlet, while the ileum end was the outlet. A peristaltic pump was used to pump the inlet with 10 µM G-Rg3r or G-Rg3s in HBSS iso-osmotic solution at a flow rate of 0.1 mL/min. The role of verapamil was assessed by adding 50 µM or 100 µM of the compound to 10 µM G-Rg3r or G-Rg3s. UPLC‒MS/MS was performed for perfused extracts collected at 60, 90, 120, 150 min time points postperfusion initiation. The formula Absorption % = (1 − Cout/Cin) × 100% was employed for the quantification of the absorption percentage; G-Rg3 concentrations at the outlet and inlet are represented by Cout and Cin, respectively.

### Cell viability assay

hEL cells were seeded in 96-well plates at a density of 1 × 10^4^ cells per well and treated with B(a)P (0, 1, 5, 10, 20, 30, 40 µM) or G-Rg3 dissolved in DMSO and then diluted with culture medium to various concentrations (0, 1, 2, 5, 10, 20 µM) for 48 h. A commercially available MTS assay kit was used, and cells were subjected to the standard protocol followed by measurement at 490 nm wavelength with a microplate reader. The cell viability levels of the B(a)P (10 µM) and G-Rg3 (0, 1, 5, 10, 20 µM) cotreatment groups were evaluated and compared with the nontreatment group according to the methods described above.

### Analysis of BPDE-DNA adduct formation

hEL cells were seeded in 6-well plates at a density of 1 × 10^5^ cells/well and treated with 10 µM B(a)P in the presence or absence of 10 µM G-Rg3. After treatment for 48 h, DNA was extracted from hEL cells using the QIAamp DNA Mini Kit according to the manufacturer’s protocol. BPDE-DNA adduct formation was detected using the BPDE-DNA adduct ELISA kit. The relative BPDE-DNA adduct levels were measured using a microplate reader for absorbance at 450 nm.

### GST activity assay

hEL cells were seeded in 96-well plates at a density of 1 × 10^4^ cells per well and treated with 10 µM B(a)P in the absence or presence of 10 µM G-Rg3 for 48 h. GST activity was measured using a commercial GST activity assay kit according to the manufacturer’s protocols. The relative activation of GST was measured by using a microplate reader for absorbance at 450 nm.

### Western blotting

hEL cells were washed using ice-cold PBS and then lysed using radioimmunoprecipitation assay buffer containing a protease inhibitor and phosphatase inhibitors. After protein quantification using a total protein assay kit, 30 µg of protein from each sample was separated by 12% SDS‒PAGE and transferred electrophoretically onto the PVDF membrane. The membranes were blocked using 5% skim milk and then incubated with primary antibodies overnight at 4 °C. After incubation with horseradish peroxidase-conjugated secondary antibody, enhanced chemiluminescent reagent was added to visualize the binding signals. The intensity of each protein band was quantified using ImageJ software.

### Statistical analysis

GraphPad Prism 7.0 software was employed for all analyses of data represented as the mean ± standard deviation. Student’s *t* test or one-way analysis of variance was utilized to evaluate the variation across the treatment groups with a *P* value < 0.05 representing statistical significance.

## Supplementary Information

Supplementary Information.

## Data Availability

All data generated or analyzed during this study are included in this published article and its supplementary information files.

## References

[1] Chen W, Zheng R, Baade PD (2016). Cancer statistics in China, 2015. CA Cancer J. Clin..

[2] Torre LA, Siegel RL, Jemal A (2016). Lung cancer statistics. Adv. Exp. Med. Biol..

[3] Hecht S (2003). Tobacco carcinogens, their biomarkers and tobacco-induced cancer. Nat. Rev. Cancer..

[4] Phillips DH, Venitt S (2012). DNA and protein adducts in human tissues resulting from exposure to tobacco smoke. Int. J. Cancer..

[5] Yan Y, Wang Y, Tan Q, Lubet RA, You M (2005). Efficacy of deguelin and silibinin on benzo(a)pyrene-induced lung tumorigenesis in A/J mice. Neoplasia.

[6] Tan W, Jinjian Lu, Huang M (2011). Anti-cancer natural products isolated from chinese medicinal herbs. Chin. Med..

[7] Yan Y, Wang Y, Tan Q (2006). Efficacy of polyphenon E, red ginseng, and rapamycin on benzo(a)pyrene-induced lung tumorigenesis in A/J mice. Neoplasia.

[8] Wang CZ, Anderson S, Du W, He TC, Yuan CS (2016). Red ginseng and cancer treatment. Chin. J. Nat. Med..

[9] Li TSC, Mazza G, Cottrell AC, Gao L (1996). Ginsenosides in roots and leaves of American ginseng. J. Agric. Food Chem..

[10] Attele AS, Wu JA, Yuan CS (1999). Ginseng pharmacology: Multiple constituents and multiple actions. Biochem. Pharmacol..

[11] Jia L, Zhao Y (2009). Current evaluation of the millennium phytomedicine-ginseng (I): Etymology, pharmacognosy, phytochemistry, market and regulations. Curr. Med. Chem..

[12] Jin ZH, Qiu W, Liu H, Jiang XH, Wang L (2018). Enhancement of oral bioavailability and immune response of Ginsenoside Rh2 by co-administration with piperine. Chin. J. Nat. Med..

[13] Sharom FJ (2008). ABC multidrug transporters: structure, function and role in chemoresistance. Pharmacogenomics.

[14] van Waterschoot RA, Lagas JS, Wagenaar E, van der Kruijssen CM, van Herwaarden AE, Song JY, Schinkel AH (2009). Absence of both cytochrome P450 3A and P-glycoprotein dramatically increases docetaxel oral bioavailability and risk of intestinal toxicity. Cancer Res..

[15] Yang K, Wu J, Li X (2008). Recent advances in the research of P-glycoprotein inhibitors. Biosci. Trends..

[16] Zhang QH, Wu CF, Duan L, Yang JY (2008). Protective effects of ginsenoside Rg3 against cyclophosphamide-induced DNA damage and cell apoptosis in mice. Arch. Toxicol..

[17] Zalewska-Ziob M, Adamek B, Kasperczyk J, Romuk E, Hudziec E (2019). Activity of antioxidant enzymes in the tumor and adjacent noncancerous tissues of non-small-cell lung cancer. Oxid. Med. Cell Longev..

[18] Unoki T, Akiyama M, Kumagai Y (2020). Nrf2 activation and its coordination with the protective defense systems in response to electrophilic stress. Int. J. Mol. Sci..

[19] Wang Y, Yao R, Gao S (2013). Chemopreventive effect of a mixture of Chinese herbs (antitumor B) on chemically induced oral carcinogenesis. Mol. Carcinog..

[20] Yang Z, Gao S, Wang J (2011). Enhancement of oral bioavailability of 20(S)-ginsenoside Rh2 through improved understanding of its absorption and efflux mechanisms. Drug Metab. Dispos..

[21] Kim BM, Kim DH, Park JH, Na HK, Surh YJ (2013). Ginsenoside Rg3 induces apoptosis of human breast cancer (MDA-MB-231) cells. J. Cancer Prev..

[22] Li Y, Yang T, Li J (2016). Inhibition of multiple myeloma cell proliferation by ginsenoside Rg3 via reduction in the secretion of IGF-1. Mol. Med. Rep..

[23] Zhang F, Li M, Wu X (2015). 20 (S)-ginsenoside Rg3 promotes senescence and apoptosis in gallbladder cancer cells via the p53 pathway. Drug Des. Devel. Ther..

[24] Chen QJ, Zhang MZ, Wang LX (2010). Gensenoside Rg3 inhibits hypoxia-induced VEGF expression in human cancer cells. Cell Physiol. Biochem..

[25] Lee SG, Kang YJ, Nam JO (2015). Anti-metastasis effects of ginsenoside Rg3 in B16F10 cells. J. Microbiol. Biotechnol..

[26] Liu TG, Huang Y, Cui DD (2009). Inhibitory effect of ginsenoside Rg3 combined with gemcitabine on angiogenesis and growth of lung cancer in mice. BMC Cancer.

[27] Wang L, Li X, Song YM (2015). Ginsenoside Rg3 sensitizes human non-small cell lung cancer cells to radiation by targeting the nuclear factor-κB pathway. Mol. Med Rep..

[28] Poon PY, Kwok HH, Yue PY (2011). Cytoprotective effect of 20(S)-Rg3 on Benzo(α)pyrene-induced DNA damage. Drug Metab. Dispos..

[29] Paek IP, Moon Y, Kim J (2006). Pharmacokinetics of a ginseng saponin metabolite compound K in rats. Biopharm. Drug Dispos..

[30] Yang Z, Wang J-R, Niu T (2012). Inhibition of P-glycoprotein leads to improved oral bioavailability of compound K, an anti-cancer metabolite of red ginseng extract produced by gut microflora. Drug Metab. Dispos..

[31] Meng Z, Zhang H, Zhao Y, Lan J, Lijun Du (2007). Transport behavior and efflux of Rg1 in rat pulmonary epithelial cells. Biomed. Chromatogr..

[32] Liang Y, Zhou Y, Zhang J (2014). Pharmacokinetic compatibility of ginsenosides and schisandra lignans in shengmai-san: From the perspective of P-Glycoprotein. PLoS ONE.

[33] Tian J, Fu F, Geng M, Jiang Y (2005). 20(S)-ginsenoside Rg3 on cerebral ischemia in rats. Neurosci. Lett..

[34] Yuan L, Lv B, Zha J, Wang W, Wang Z (2014). Basal and benzo[a]pyrene-induced expression profile of phase I and II enzymes and ABC transporter mRNA in the early life stage of Chinese rare minnows (Gobiocypris rarus). Ecotoxicol. Env. Saf..

[35] Cai Y, Pan L, Miao J (2016). In vitro study of the effect of metabolism enzymes on benzo(a)pyrene-induced DNA damage in the scallop Chlamys farreri. Environ. Toxicol Pharm..

[36] Lim J, Ortiz L, Nakamura BN, Hoang YD, Banuelos J, Flores VN, Chan JY, Luderer U (2015). Effects of deletion of the transcription factor Nrf2 and benzo [a]pyrene treatment on ovarian follicles and ovarian surface epithelial cells in mice. Reprod. Toxicol..

[37] Shen Y, Liu X, Shi J, Wu X (2019). Involvement of Nrf2 in myocardial ischemia and reperfusion injury. Int. J. Biol. Macromol..

[38] Leinonen HM, Kansanen E, Pölönen P, Heinäniemi M, Levonen AL (2014). Role of the Keap1-Nrf2 pathway in cancer. Adv. Cancer Res..

