# Supplementary material for: The preventive role of the red gingeng ginsenoside Rg3 in the treatment of lung tumorigenesis induced by benzo(a)pyrene
Source: Sci Rep. 2023 Mar 20;13:4528. doi: 10.1038/s41598-023-31710-9 (PMC10027881; doi:10.1038/s41598-023-31710-9)

Figure 1A The experimental design for the treatment of lung oncogenesis in A/J mice by the red ginseng extracts.


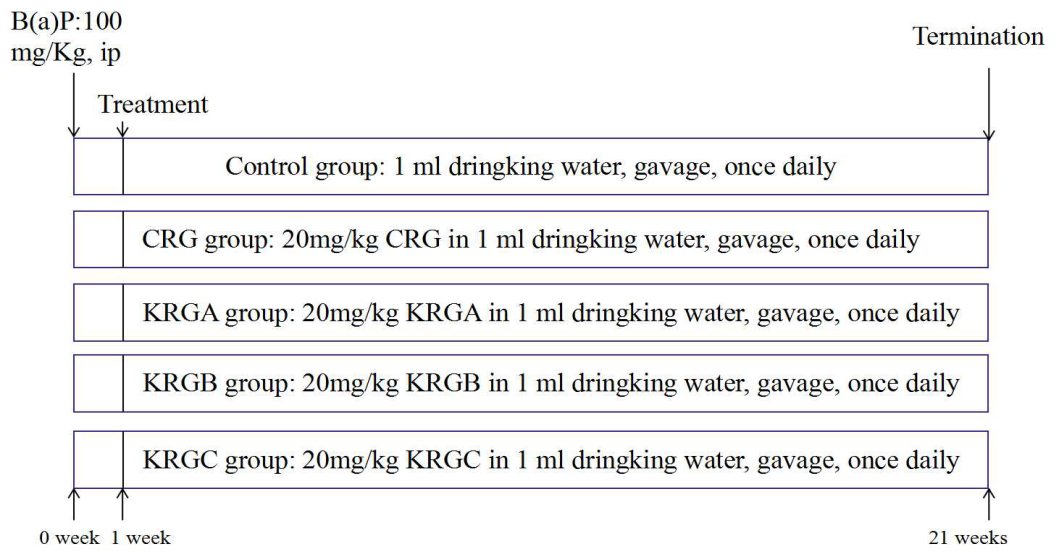


CRG: Chinese red ginseng, KRGA: Korean red gingseng A, KRGB: Korean red ginseng B, KRGC: Korean red ginseng C.

Figure 1B: Gross lung tumors in the murine model

**
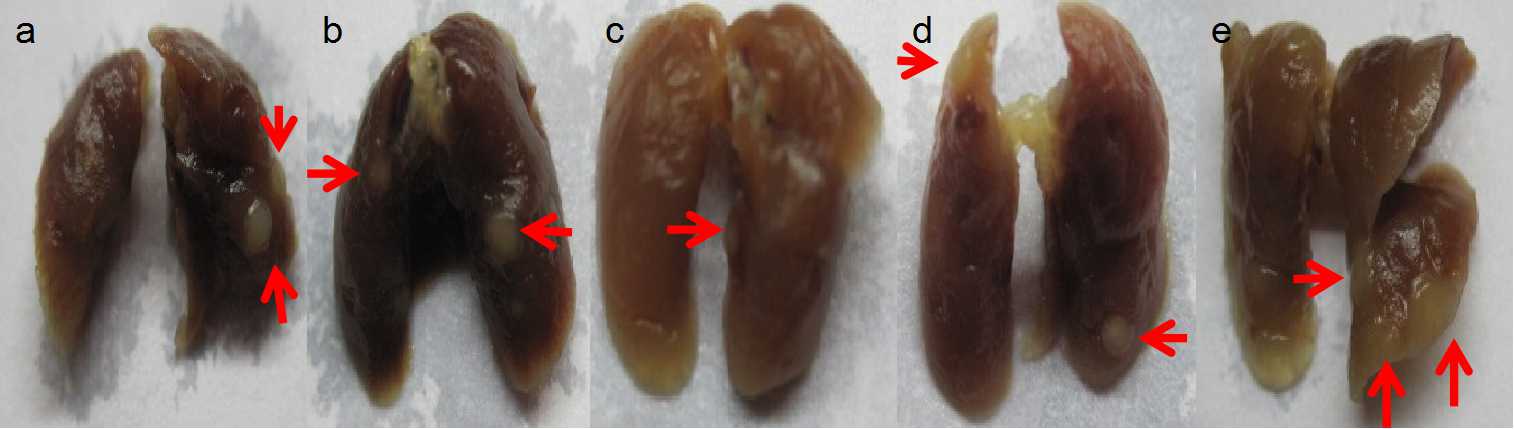
**

Tumors are indicated by arrows. a: CRG group. b: KRGA group. c: KRGB group. d: KRGC group. e: Control group.

Figure 1C: Lung tumors illustrated by light photomicrographs


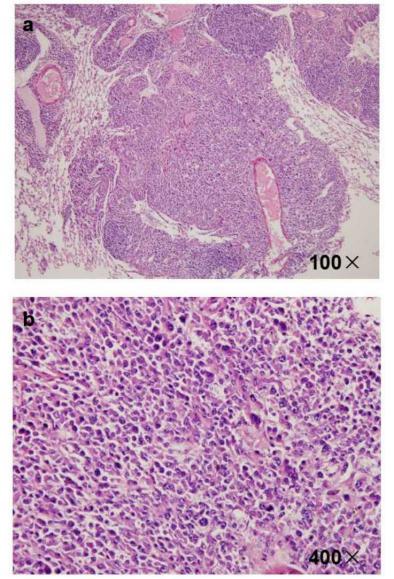


The magnification is a: 100. b: 400.

Figure 1D: The tumor load in red ginseng extract groups.


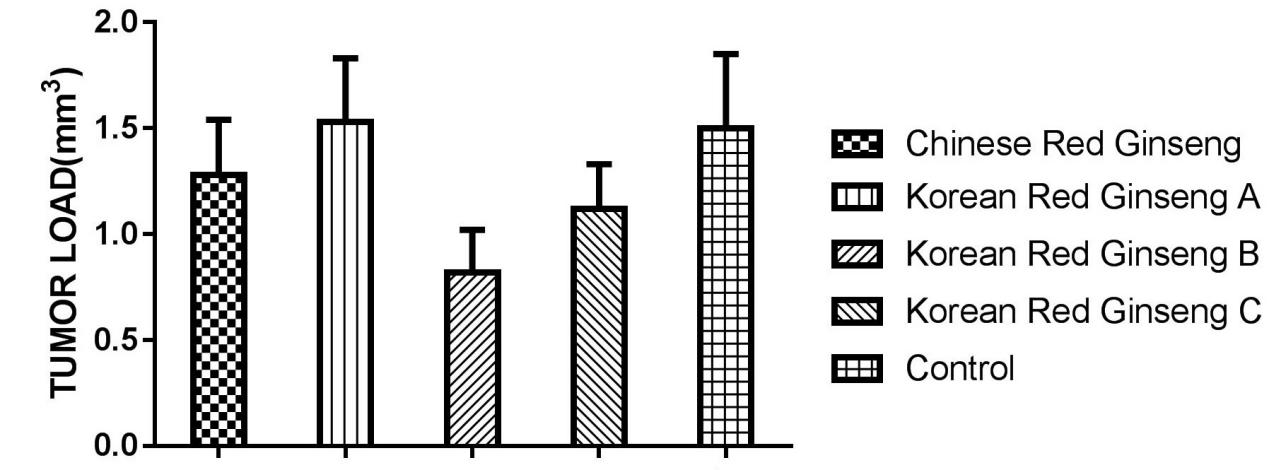


The mean ± SD is represented by error bars

Figure 1E: The plasma levels of the liver enzyme ALT in each group.


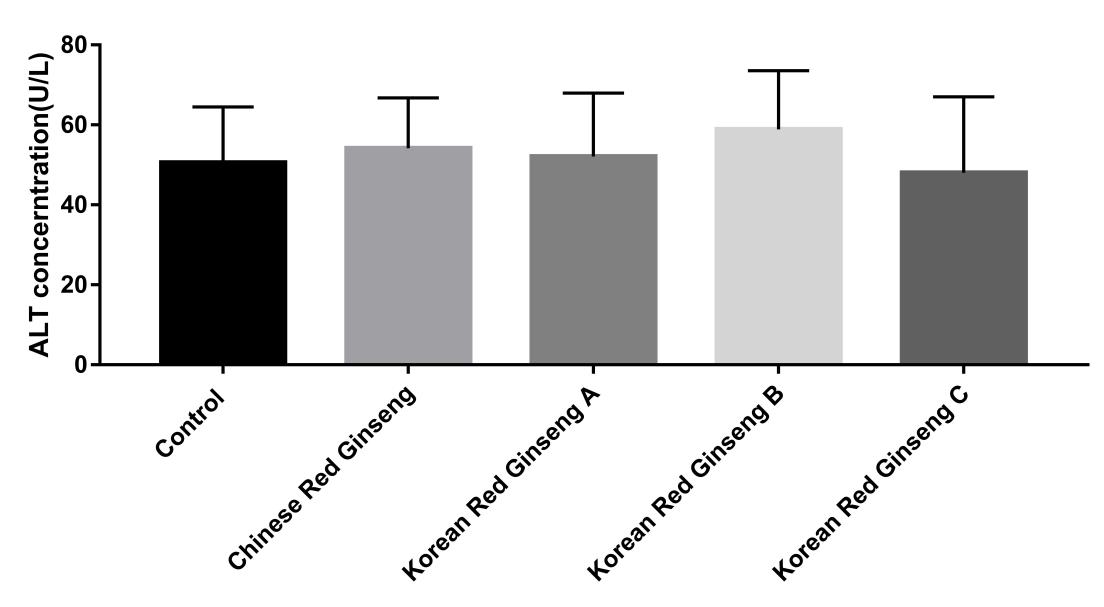


The mean ± SD is represented by error bars

Figure 1F: The plasma levels of the kidney enzyme Cr in each group.


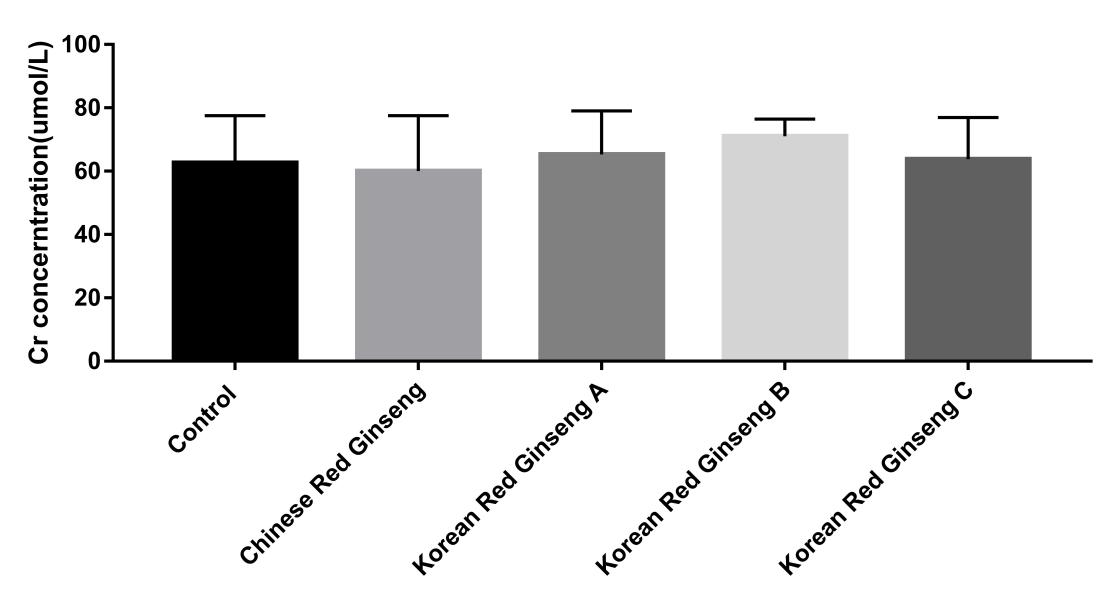


The mean ± SD is represented by error bars

Figure 2A: UPLC-MS/MS chromatogram of four red ginseng extracts


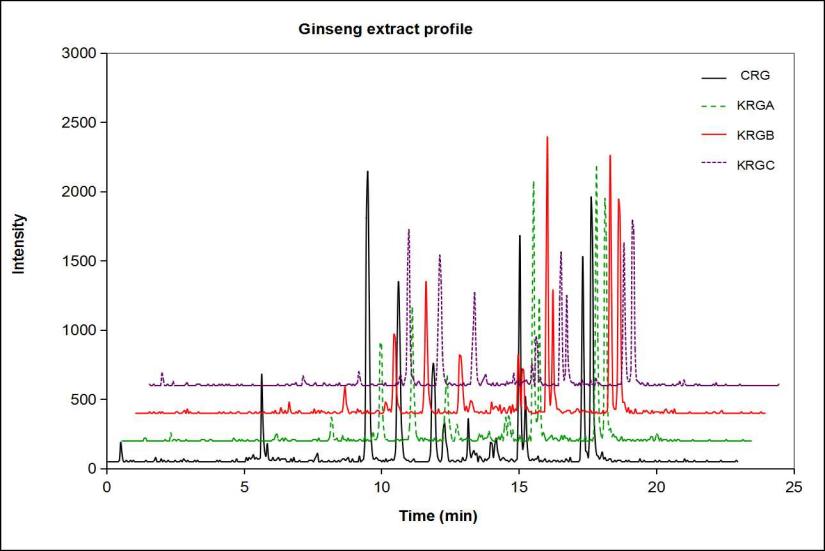


CRG: Chinese red ginseng, KRGA: Korean red ginseng A, KRGB: Korean red ginseng B, KRGC: Korean red ginseng C.

Figure 2B: Estimation of total ginsenosides in the drinking water of each ginseng extracts


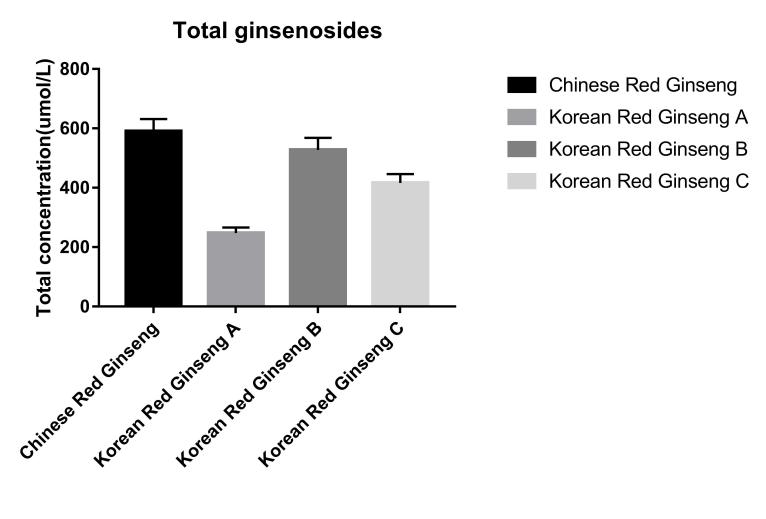


The mean ± SD represents the data of five independent experiments are presented

Figure 2C: Detection of individual ginsenosides in the drinking water of the ginseng extracts


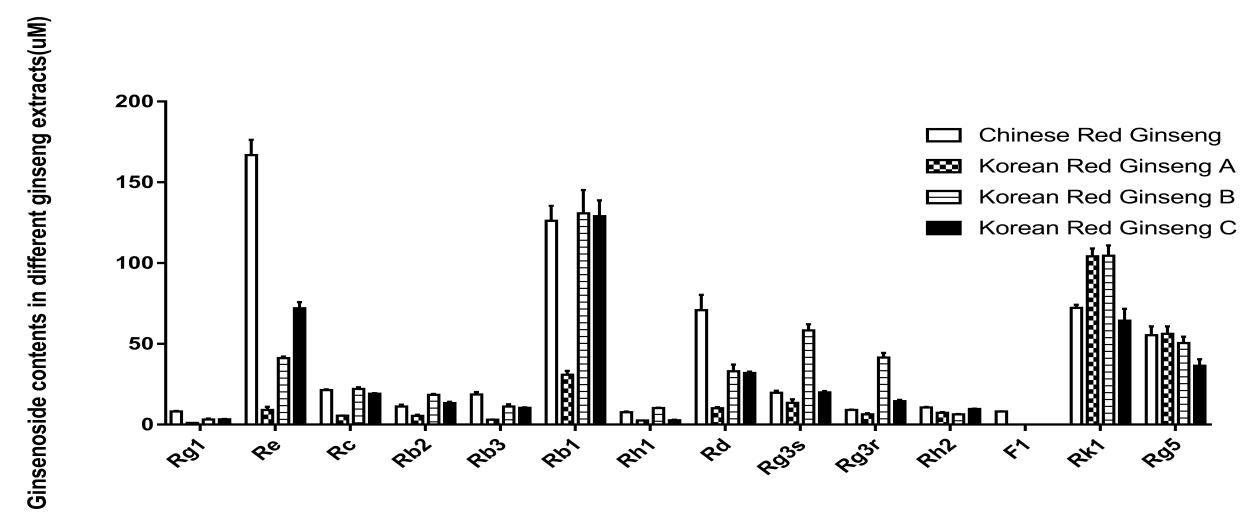


The mean ± SD represents the data of five independent experiments are presented

Figure 2D: Structures of the ginsenosides Rg3r and Rg3s stereoisomers.


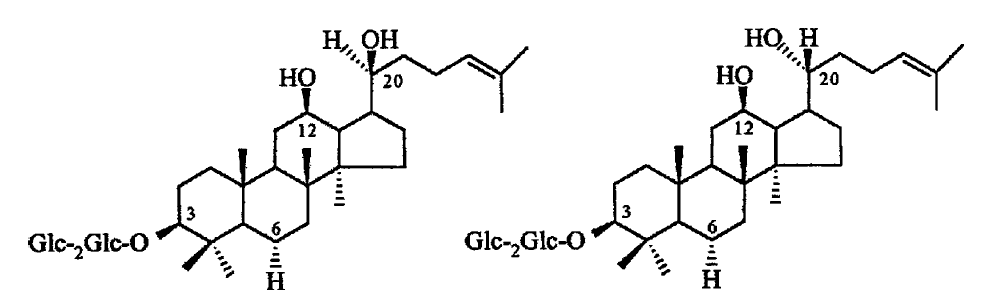


Figure3A: Pa-b values of Rg3r groups in the Caco-2 monolayer transport model.


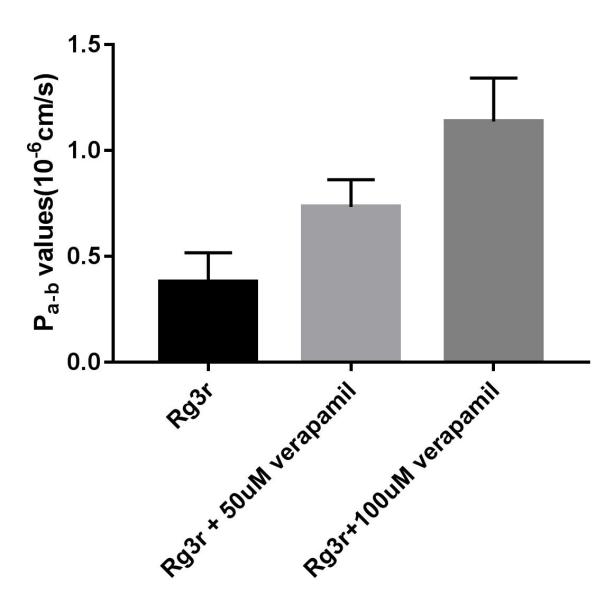


The mean ± SD represents the data of five independent experiments are presented

Figure3B: Pa-b values of Rg3s groups in the Caco-2 monolayer transport model.


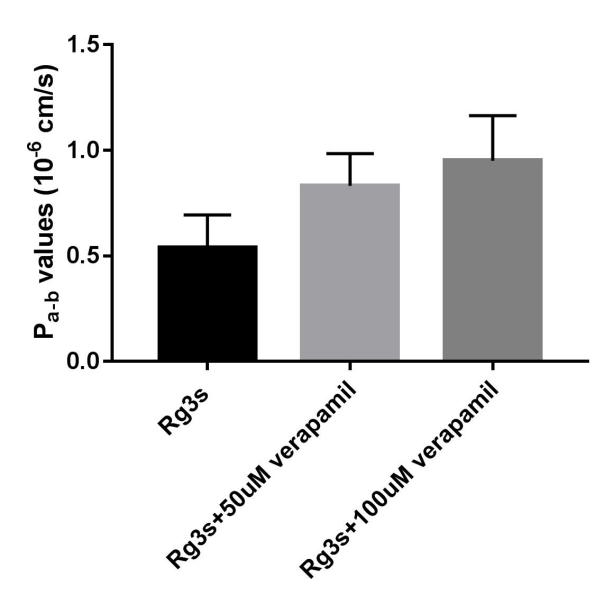


The mean ± SD represents the data of five independent experiments are presented

Figure3C: Pb-a values of Rg3r groups in the Caco-2 monolayer.


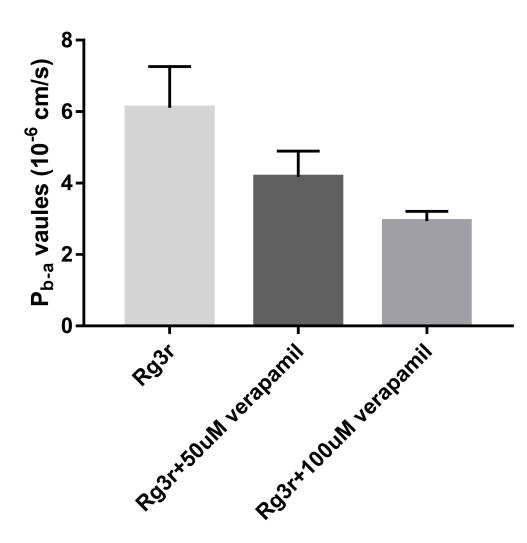


The mean ± SD represents the data of five independent experiments are presented

Figure3D: Pb-a values of Rg3s groups in the Caco-2 monolayer.


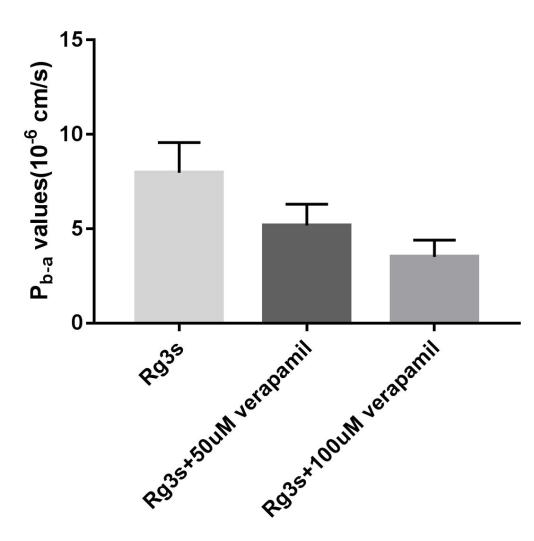


The mean ± SD represents the data of five independent experiments are presented

Figure3E: Efflux ratio of Rg3r groups in the Caco-2 monolayer


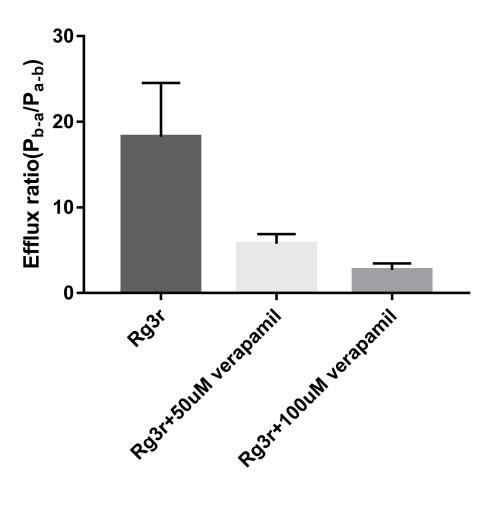


The mean ± SD represents the data of five independent experiments are presented

Figure3F: Efflux ratio of Rg3s groups in the Caco-2 monolayer


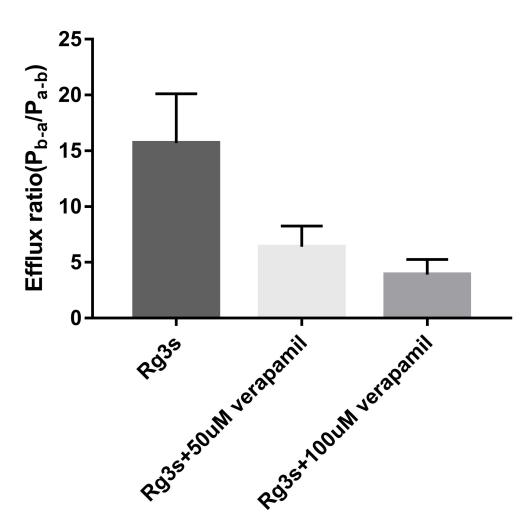


The mean ± SD represents the data of five independent experiments are presented

Figure3G: Intestinal absorption percentages of Rg3r in the rat perfusion assay


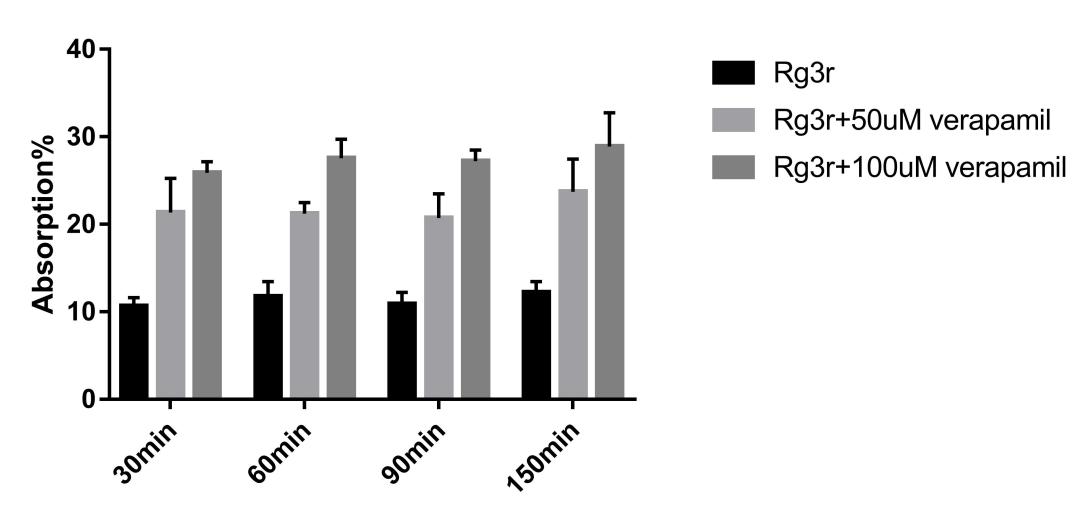


The mean ± SD represents the data of five independent experiments are presented

Figure3H: Intestinal absorption percentages of Rg3s in the rat perfusion assay


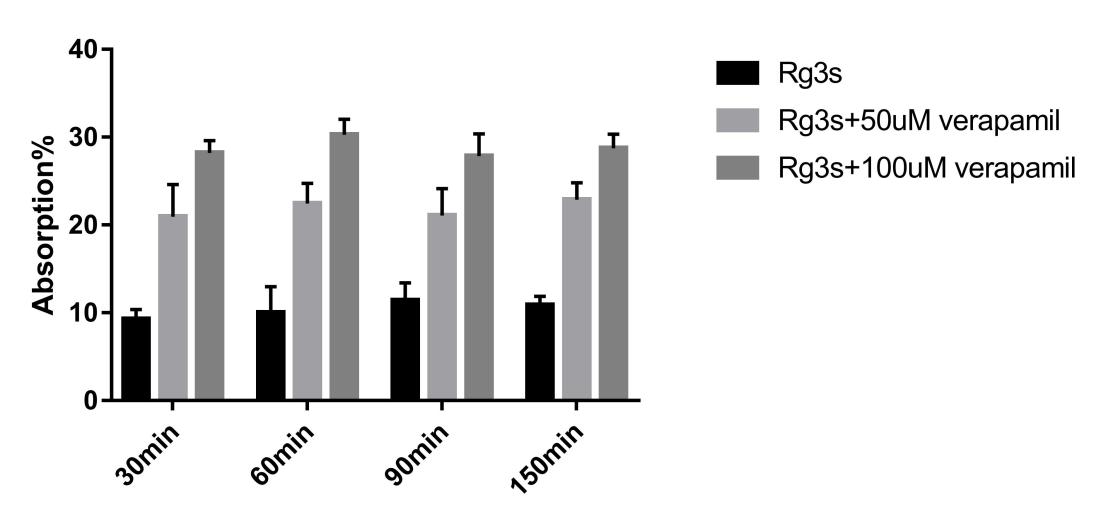


The mean ± SD represents the data of five independent experiments are presented

Figure4A: Experimental design for the tumor load impacted by Rg3


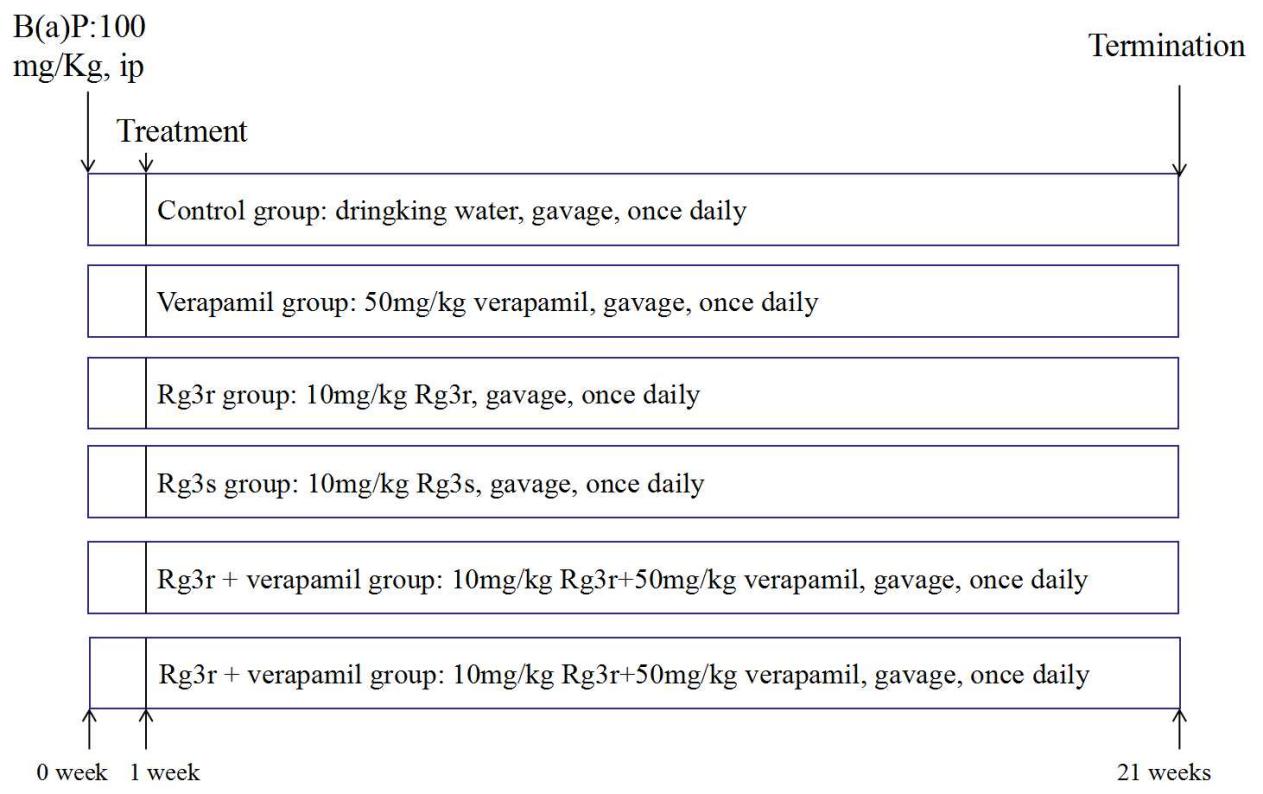


Figure 4B: Gross tumors in the murine model


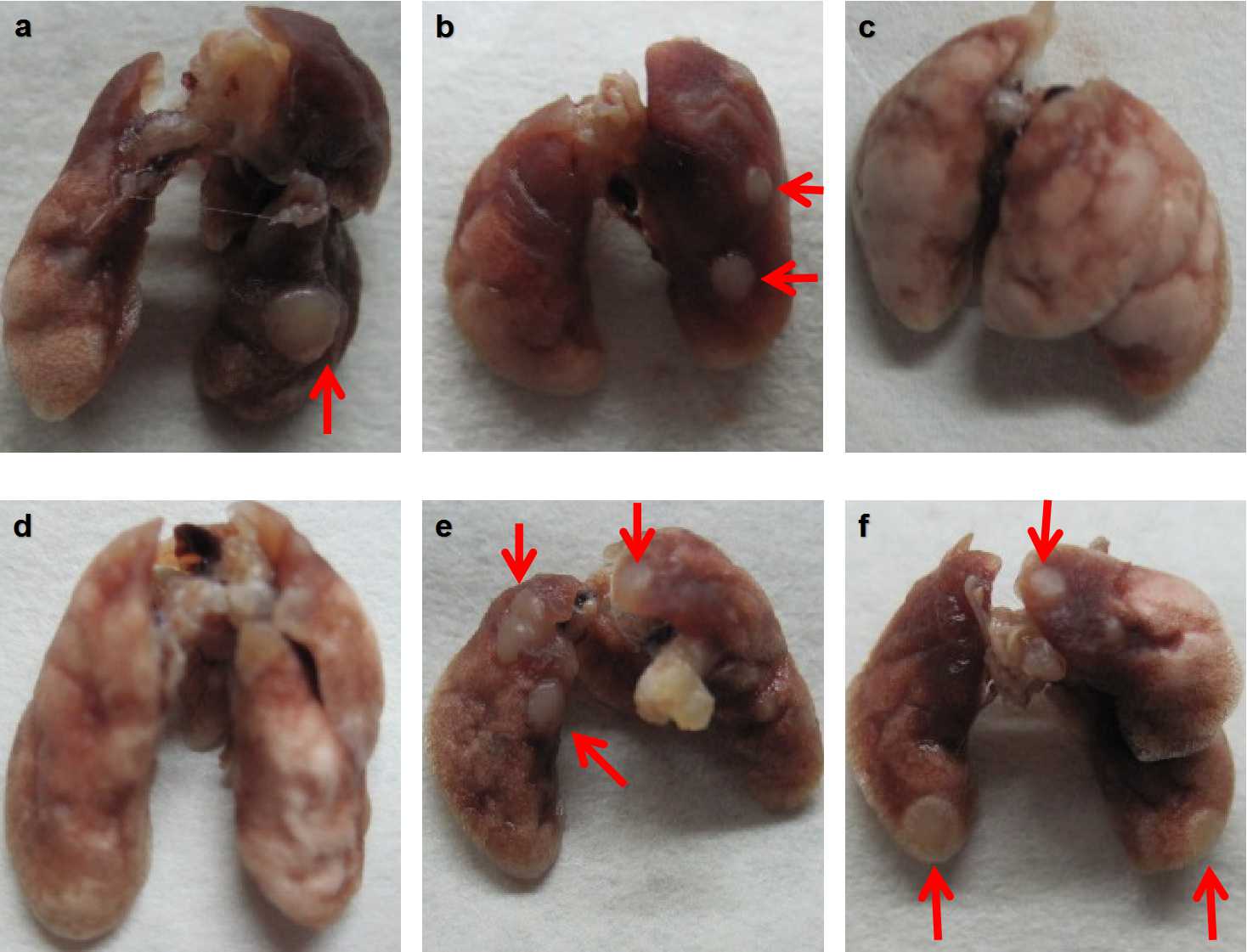


Tumors are indicated by arrows. a: Rg3r. b: Rg3s. c: Rg3r combined with verapamil. d: Rg3s combined with verapamil. e: verapamil. f: Control.

Figure 4C: Light photomicrographs of tumors.


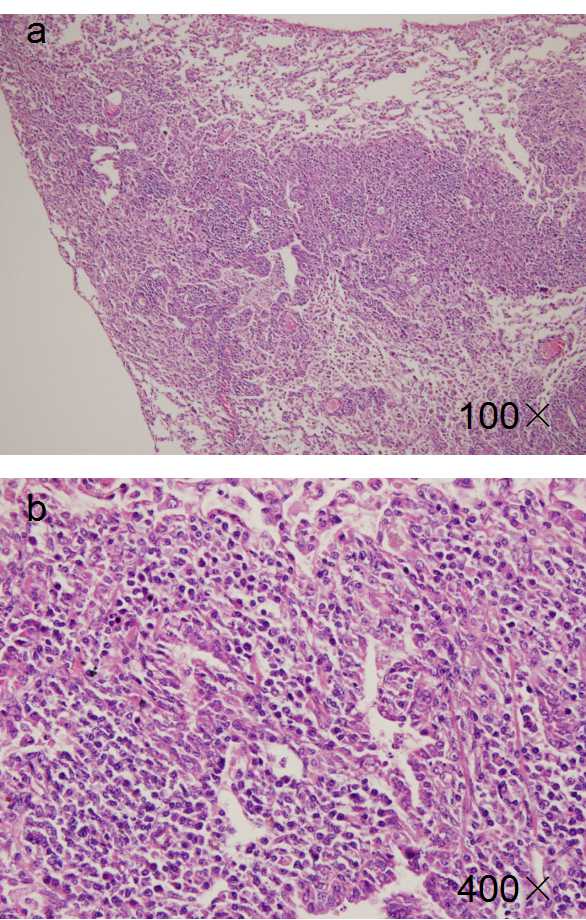


Light photomicrographs of tumors with magnification at. a: 100X. b: 400X.

Figure 4D: Rg3+verapamil impact on tumor load in A/J mice.


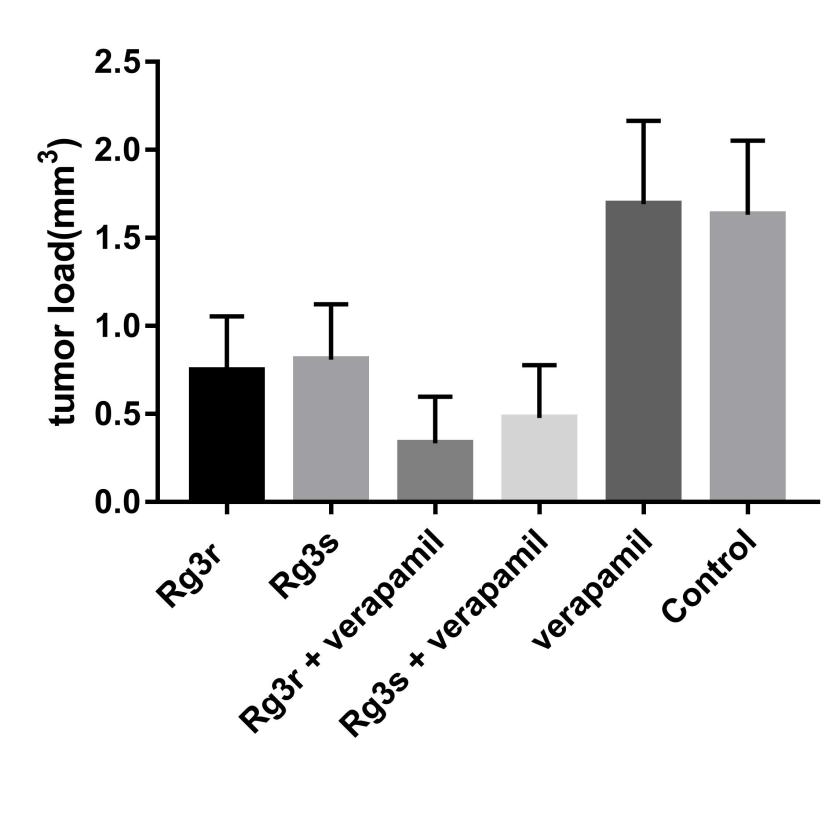


The mean ± SD is represented by error bars of triplicate assays.

Figure 4E: The plasma levels of the liver enzyme ALT.


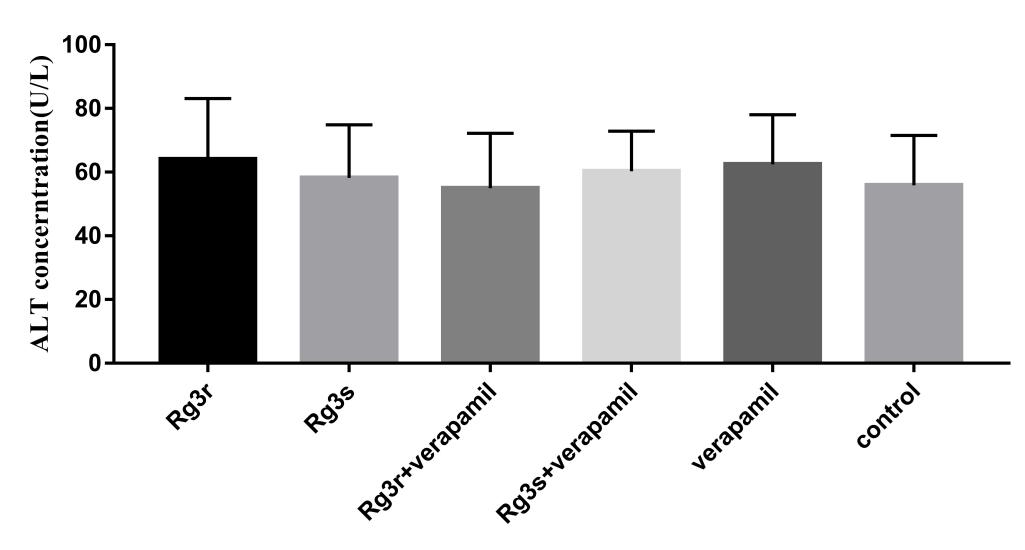


The mean ± SD is represented by error bars of triplicate assays.

Figure 4F: The plasma levels of the kidney enzyme Cr.


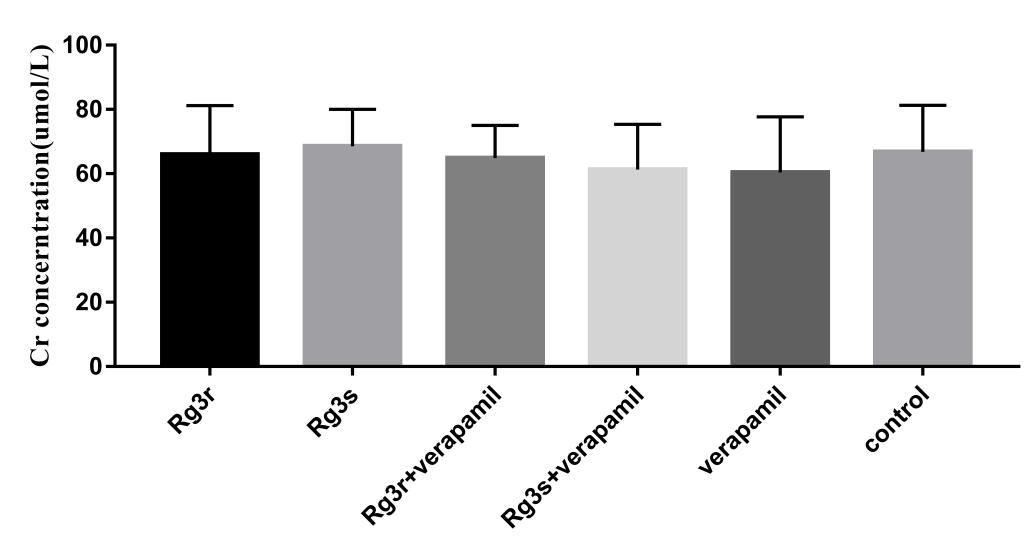


The mean ± SD is represented by error bars of triplicate assays.

Figure 4G: Levels of Rg3r or Rg3s in the plasma of the indicated groups


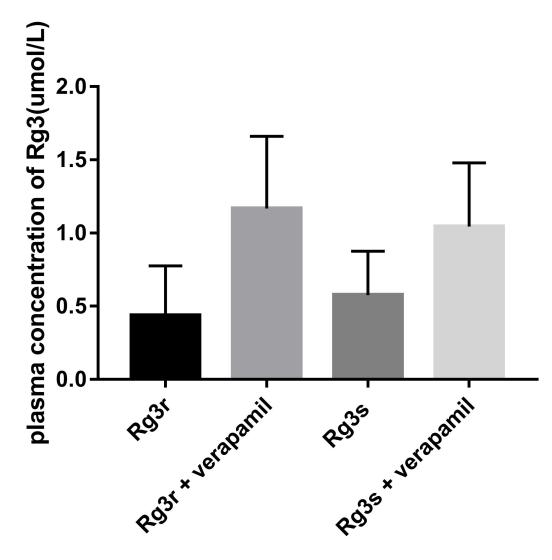


The mean ± SD is represented by error bars of triplicate assays.

Figure 4H: Levels of Rg3r or Rg3s in the intestine of the indicated groups


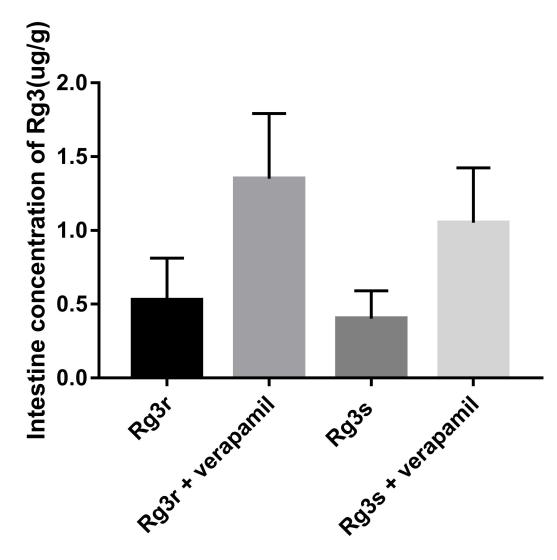


The mean ± SD is represented by error bars of triplicate assays.

Figure 5A Cell viability of hEL cells treated with B(a)P


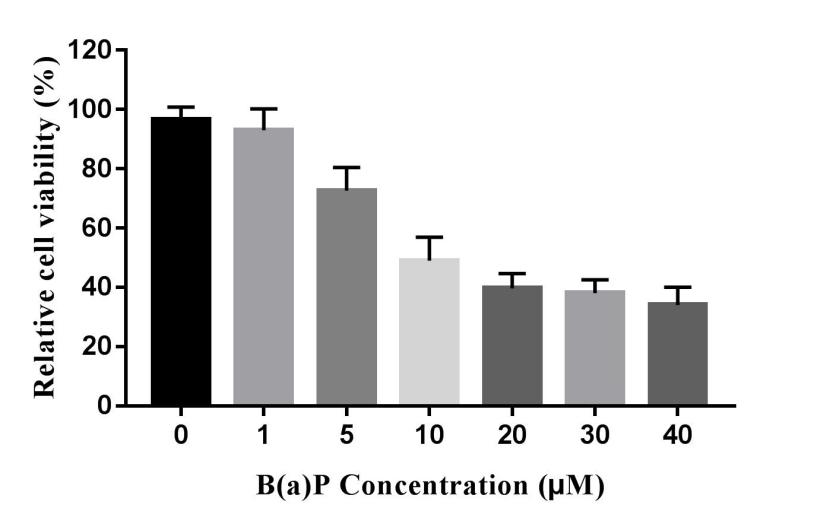


The mean ± SD is represented by error bars of triplicate assays

Figure 5B: Cell viability of hEL cells treated with Rg3r.


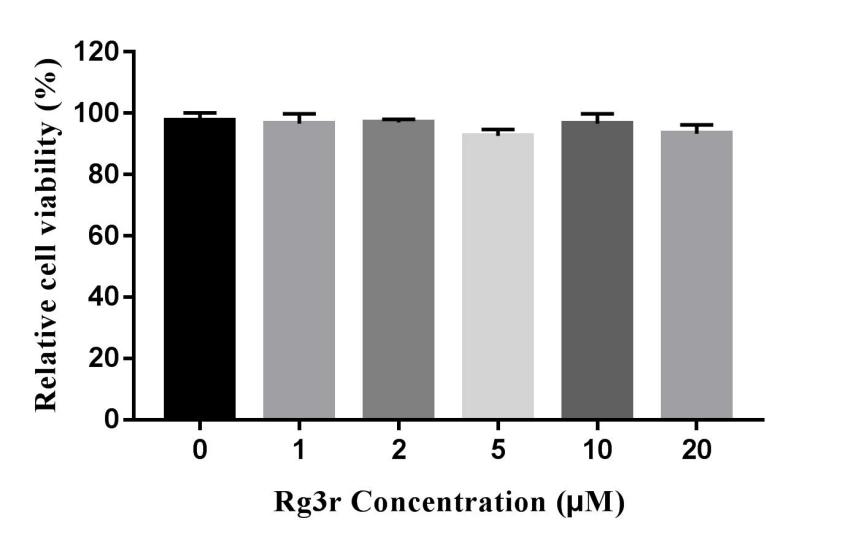


The mean ± SD is represented by error bars of triplicate assays

Figure 5C: Cell viability of hEL cells treated with Rg3s


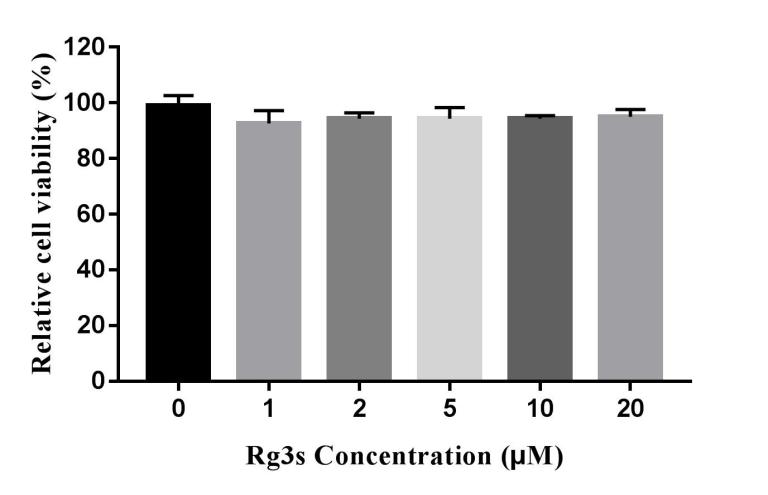


The mean ± SD is represented by error bars of triplicate assays

Figure5D: Cell viability of hEL cells treated with B(a)P and Rg3r


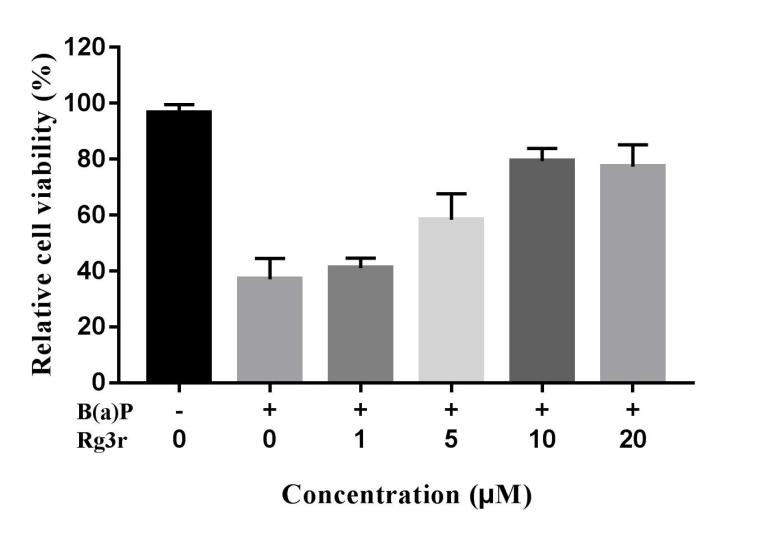


The mean ± SD is represented by error bars of triplicate assays

Figure 5E: Cell viability of hEL cells treated with B(a)P and Rg3s


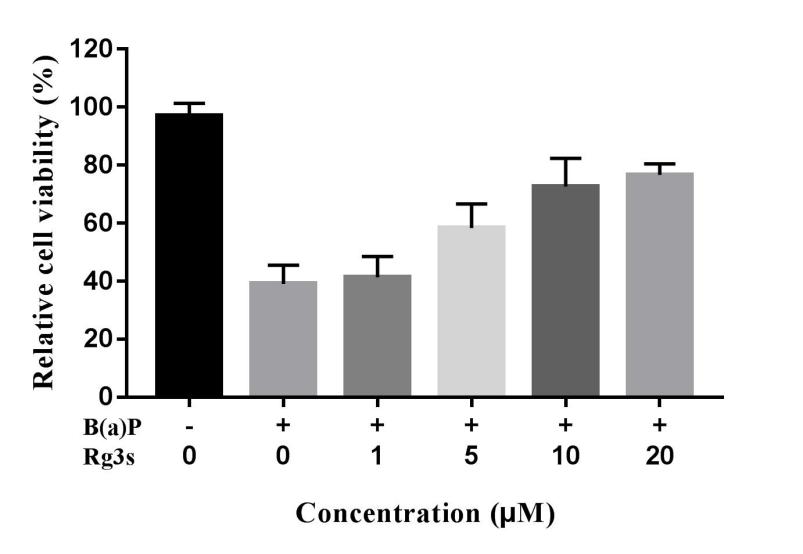


The mean ± SD is represented by error bars of triplicate assays

Figure5F: BPDE-DNA adduct levels of hEL cells treated with B(a)P and Rg3


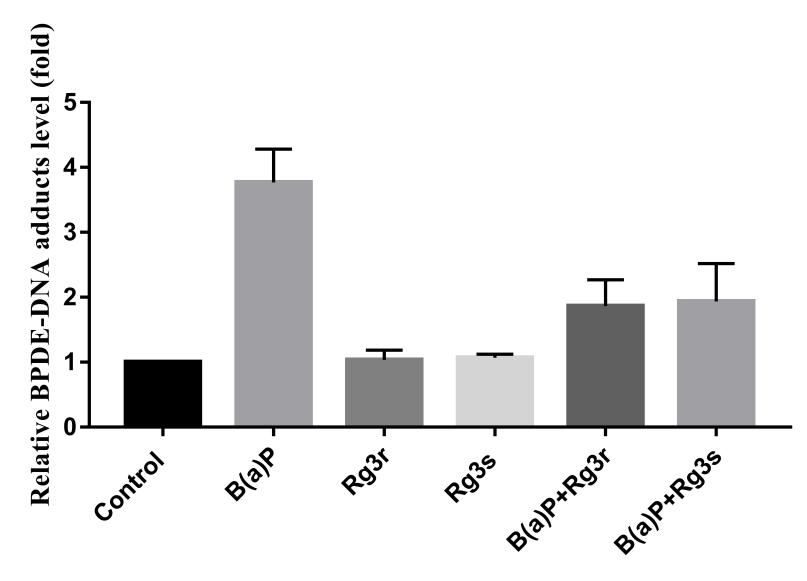


The mean ± SD is represented by error bars of triplicate assays

Figure 6A : GST expression was detected by western blotting

Control B(a)P Rg3 Rg3+B(a)P


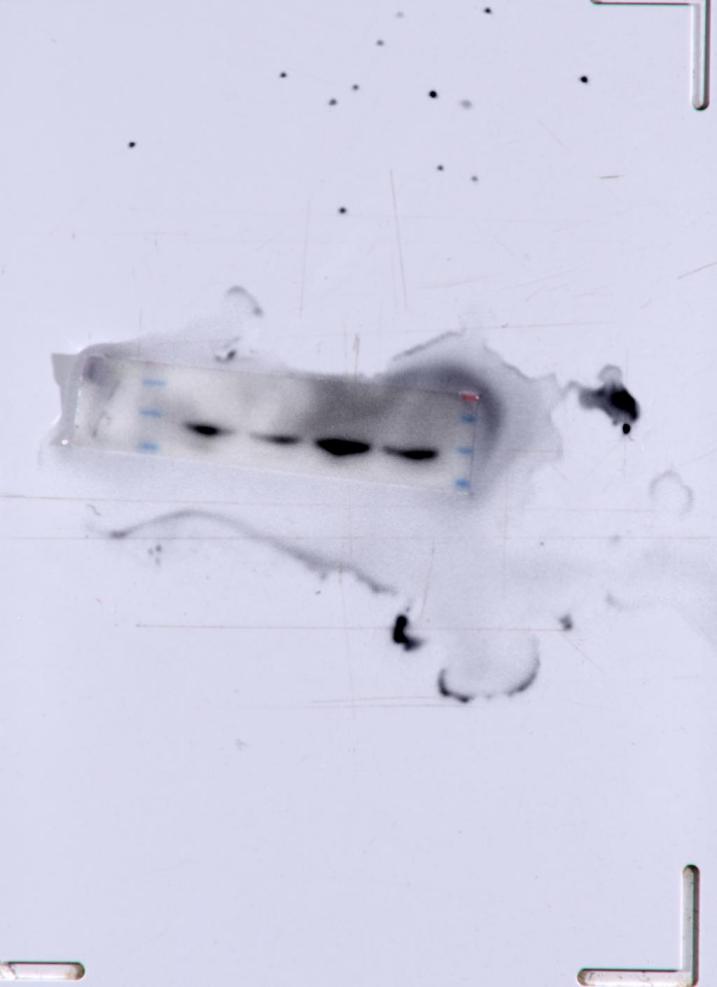


a (GST)


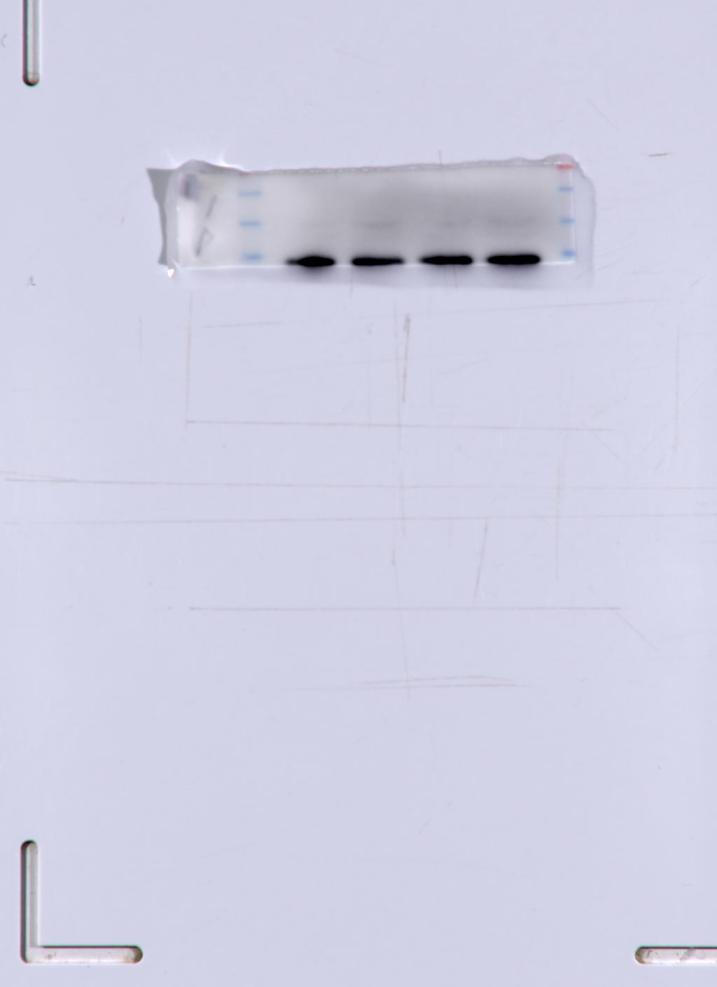


b (β-actin)


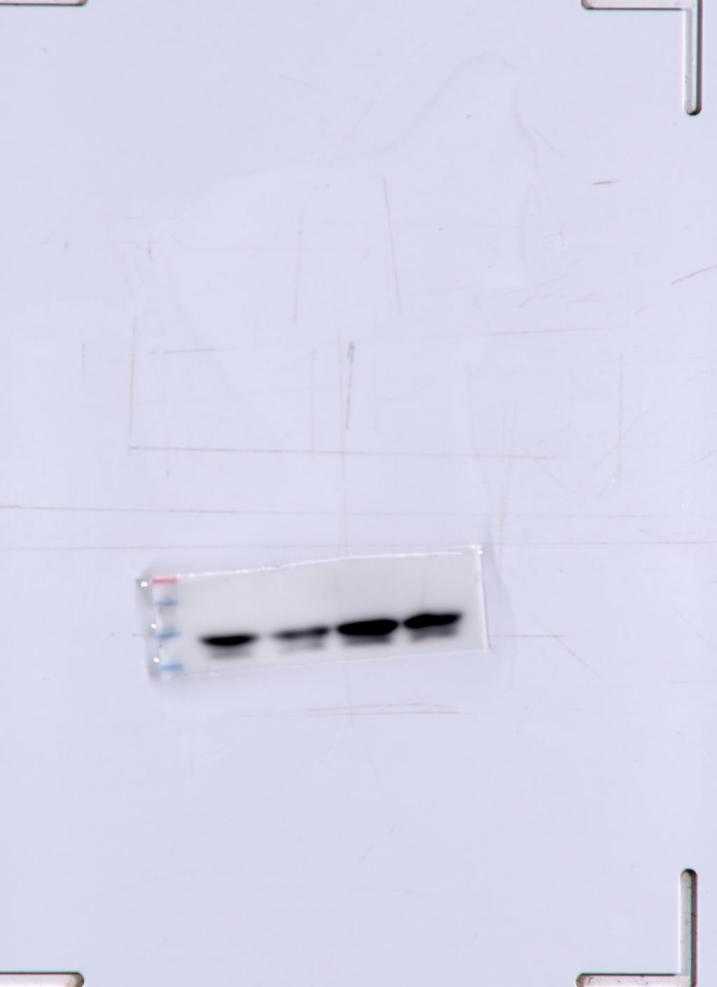


c (GST)


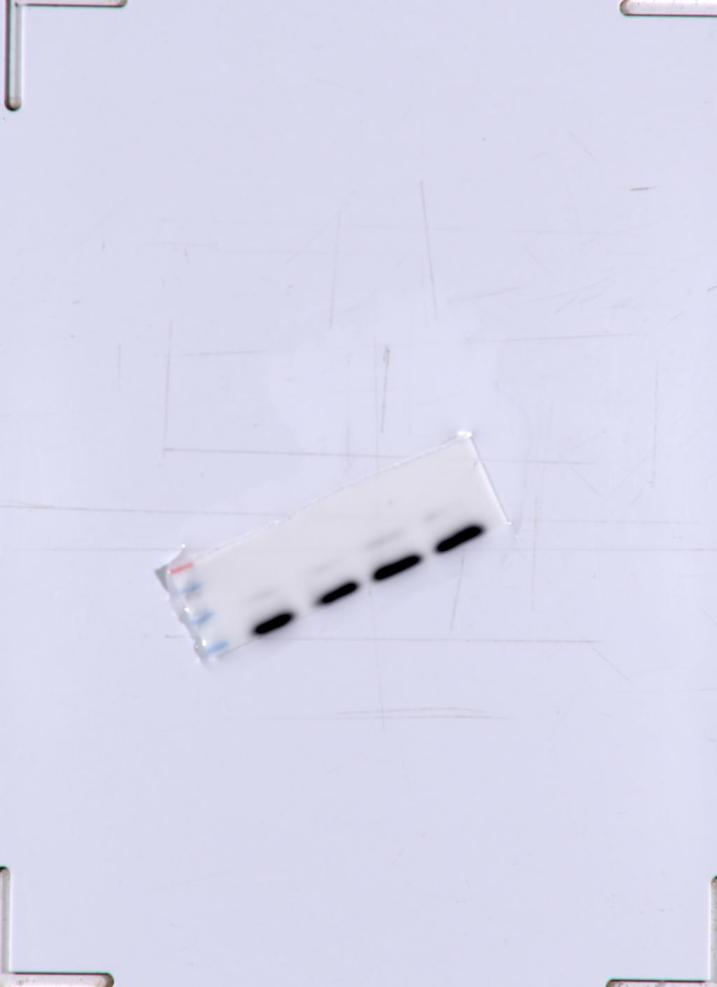


d (β-actin)

The raw picture of the western blotting of GST expression: a: GST expression in cells treated with Rg3r. b: β-actin in cells treated with Rg3r. c: GST expression in cells treated with Rg3s. d: β-actin in cells treated with Rg3s. The line from left to right indicted: Control, B(a)P, Rg3, and Rg3+B(a)P group.

Figure 6B: Quantitative GST expression in hEL cells treated with B(a)P and Rg3r.


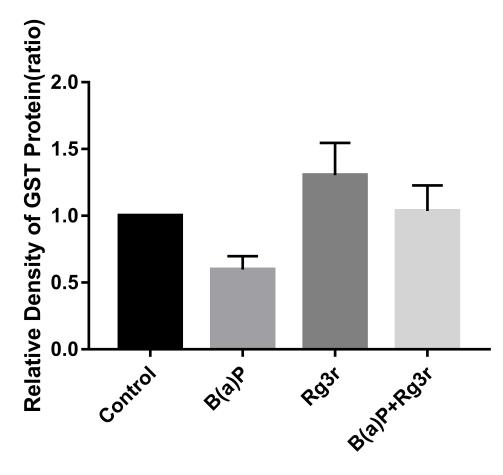


The mean ± SD is represented by error bars of triplicate assays

Figure6C: Quantitative GST expression in hEL cells treated with B(a)P and Rg3s.


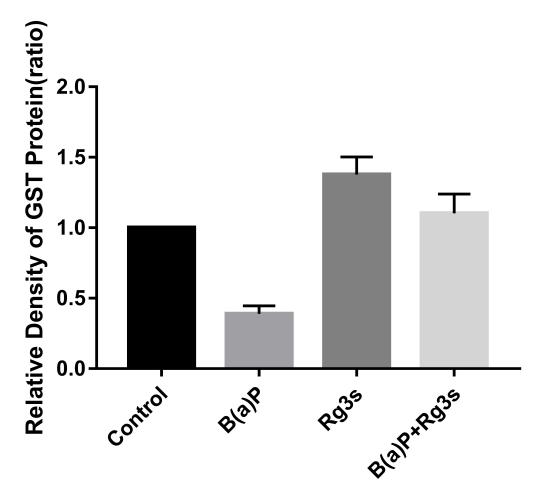


The mean ± SD is represented by error bars of triplicate assays

Figure6D: GST activity of hEL cells treated with B(a)P and Rg3.


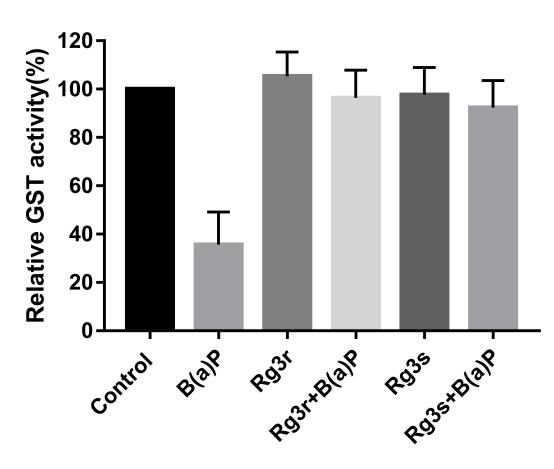


The mean ± SD is represented by error bars of triplicate assays

Figure6E: Nrf2 expression detection by western blotting


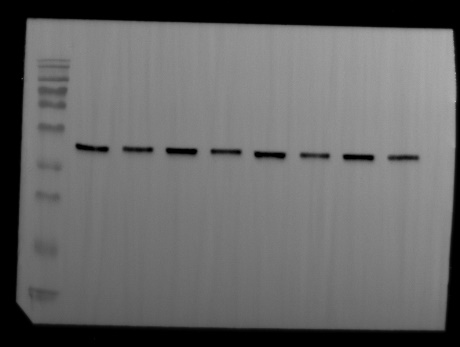


a (NRF)


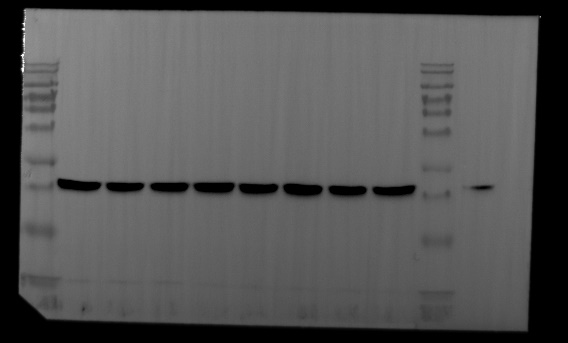


b (β-actin)

The raw picture of the western blotting of NRF expression: a: NRF expression in cells treated with Rg3r and Rg3s b: β-actin in cells treated with Rg3r and Rg3s. The left 4 lines indicated Rg3r group: Control, B(a)P, Rg3r, and Rg3r+B(a)P group. The right 4 lines indicated Rg3s group: Control, B(a)P, Rg3s, and Rg3s+B(a)P group.

Figure6F: Quantitative Nrf2 expression in hEL cells treated with B(a)P and Rg3r.


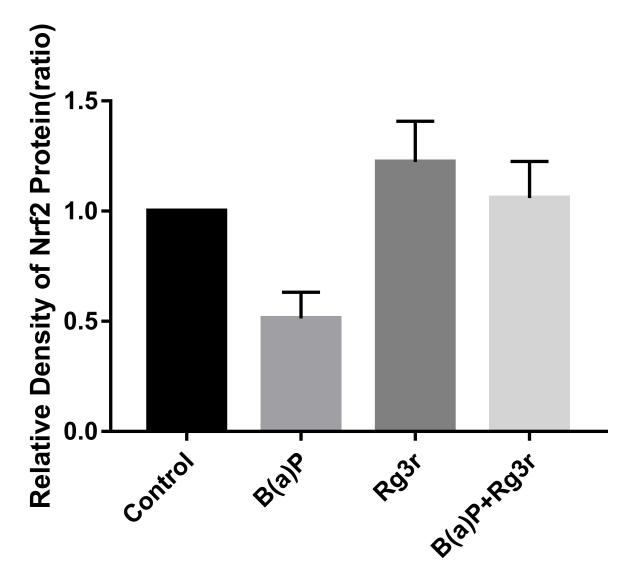


The mean ± SD is represented by error bars of triplicate assays

Figure6G: Quantitative Nrf2 expression in hEL cells treated with B(a)P and Rg3s.


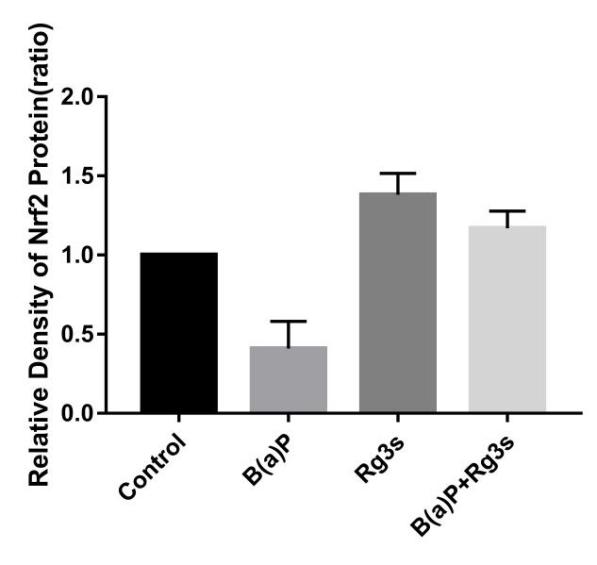


The mean ± SD is represented by error bars of triplicate assays

Figure7A: The knockdown of Nrf2 was confirmed by western blotting.

Control siNrf2 siNrf2


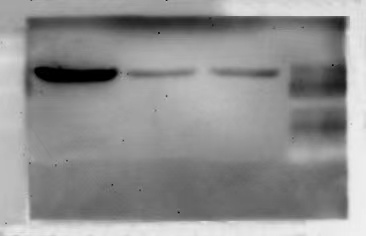


a (NRF)


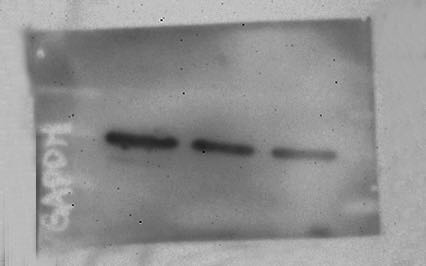


b (GAPDH)

The raw picture of the western blotting of NRF expression.a: NRF expression,b: GAPDH. The line from left to right indicted: siControl, siNRF2, siNRF2. The third ( right-most) line is the repeated siNRF2 group, and not showed in the manuscript.

Figure 7B: Quantification of the Nrf2 band intensity


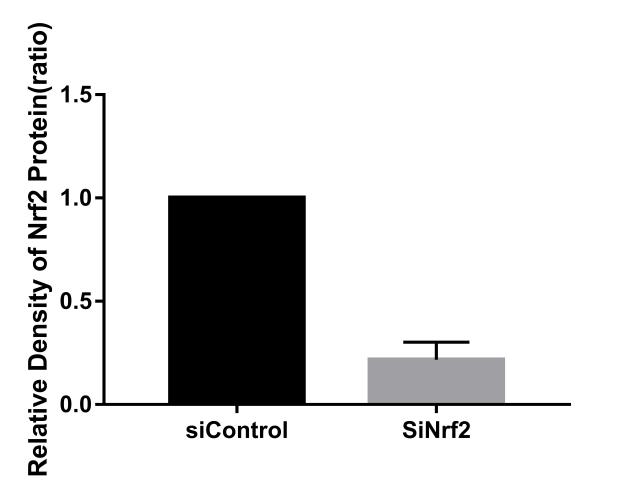


Figure 7C: Effect of knockdown of Nrf2 on BPDE-DNA adduct levels in hEL cells treated with B(a)P and Rg3r.


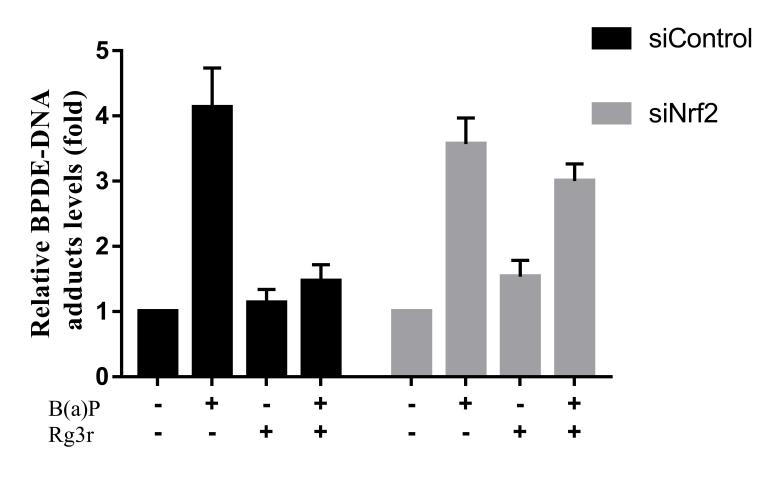


Figure 7D: Effect of knockdown of Nrf2 on BPDE-DNA adduct levels in hEL cells treated with B(a)P and Rg3s.


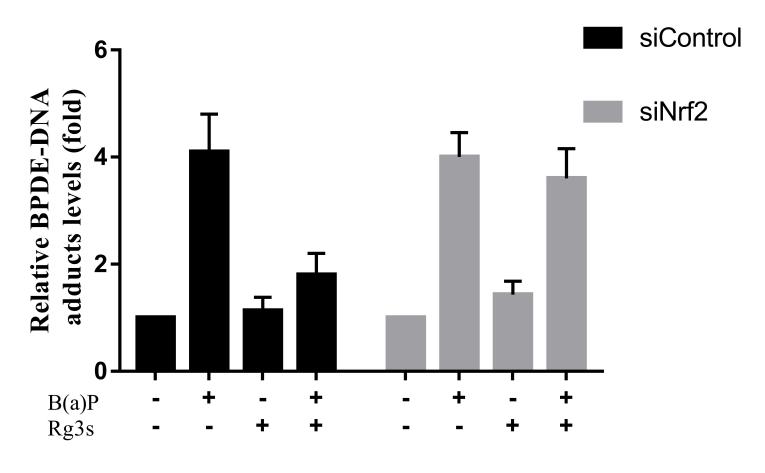

Supplement: Supplementary file 1 — Supplementary Information. [file 41598_2023_31710_MOESM1_ESM.docx]
